# Assessing the Short-Term Efficacy of Digital Cognitive Behavioral Therapy for Insomnia With Different Types of Coaching: Randomized Controlled Comparative Trial

**DOI:** 10.2196/51716

**Published:** 2024-08-07

**Authors:** Wai Sze Chan, Wing Yee Cheng, Samson Hoi Chun Lok, Amanda Kah Mun Cheah, Anna Kai Win Lee, Albe Sin Ying Ng, Tobias Kowatsch

**Affiliations:** 1 Department of Psychology The University of Hong Kong Hong Kong China (Hong Kong); 2 Institute for Implementation Science in Health Care University of Zurich Zurich Switzerland; 3 School of Medicine University of St.Gallen St. Gallen Switzerland; 4 Centre for Digital Health Interventions, Department of Management, Technology, and Economics Eidgenössische Technische Hochschule Zürich Zurich Switzerland

**Keywords:** insomnia, cognitive behavioral therapy, digital intervention, mobile health, mHealth, chatbot-based coaching, human support, mobile phone

## Abstract

**Background:**

Digital cognitive behavioral therapy for insomnia (dCBTi) is an effective intervention for treating insomnia. The findings regarding its efficacy compared to face-to-face cognitive behavioral therapy for insomnia are inconclusive but suggest that dCBTi might be inferior. The lack of human support and low treatment adherence are believed to be barriers to dCBTi achieving its optimal efficacy. However, there has yet to be a direct comparative trial of dCBTi with different types of coaching support.

**Objective:**

This study examines whether adding chatbot-based and human coaching would improve the treatment efficacy of, and adherence to, dCBTi.

**Methods:**

Overall, 129 participants (n=98, 76% women; age: mean 34.09, SD 12.05 y) whose scores on the Insomnia Severity Index [ISI] were greater than 9 were recruited. A randomized controlled comparative trial with 5 arms was conducted: dCBTi with chatbot-based coaching and therapist support (dCBTi-therapist), dCBTi with chatbot-based coaching and research assistant support, dCBTi with chatbot-based coaching only, dCBTi without any coaching, and digital sleep hygiene and self-monitoring control. Participants were blinded to the condition assignment and study hypotheses, and the outcomes were self-assessed using questionnaires administered on the web. The outcomes included measures of insomnia (the ISI and the Sleep Condition Indicator), mood disturbances, fatigue, daytime sleepiness, quality of life, dysfunctional beliefs about sleep, and sleep-related safety behaviors administered at baseline, after treatment, and at 4-week follow-up. Treatment adherence was measured by the completion of video sessions and sleep diaries. An intention-to-treat analysis was conducted.

**Results:**

Significant condition-by-time interaction effects showed that dCBTi recipients, regardless of having any coaching, had greater improvements in insomnia measured by the Sleep Condition Indicator (*P*=.003; *d*=0.45) but not the ISI (*P*=.86; *d*=–0.28), depressive symptoms (*P*<.001; *d*=–0.62), anxiety (*P*=.01; *d*=–0.40), fatigue (*P*=.02; *d*=–0.35), dysfunctional beliefs about sleep (*P*<.001; *d*=–0.53), and safety behaviors related to sleep (*P*=.001; *d*=–0.50) than those who received digital sleep hygiene and self-monitoring control. The addition of chatbot-based coaching and human support did not improve treatment efficacy. However, adding human support promoted greater reductions in fatigue (*P*=.03; *d*=–0.33) and sleep-related safety behaviors (*P*=.05; *d*=–0.30) than dCBTi with chatbot-based coaching only at 4-week follow-up. dCBTi-therapist had the highest video and diary completion rates compared to other conditions (video: 16/25, 60% in dCBTi-therapist vs <3/21, <25% in dCBTi without any coaching), indicating greater treatment adherence.

**Conclusions:**

Our findings support the efficacy of dCBTi in treating insomnia, reducing thoughts and behaviors that perpetuate insomnia, reducing mood disturbances and fatigue, and improving quality of life. Adding chatbot-based coaching and human support did not significantly improve the efficacy of dCBTi after treatment. However, adding human support had incremental benefits on reducing fatigue and behaviors that could perpetuate insomnia, and hence may improve long-term efficacy.

**Trial Registration:**

ClinicalTrials.gov NCT05136638; https://www.clinicaltrials.gov/study/NCT05136638

## Introduction

### Background

Insomnia disorder is the most common sleep-wake disorder. It is characterized by difficulty initiating or maintaining sleep despite having adequate opportunities to sleep or having nonrestorative sleep, not explained by other sleep disorders [1,2]. The prevalence of insomnia disorder ranges from 2.3% to 25.5% globally [3] and was heightened during the COVID-19 pandemic, affecting up to one-third of the general population [4-6]. Insomnia is associated with high societal and economic costs resulting from health care utilization, work absenteeism, and lost productivity [7,8]. In the United States, the estimated annual cost of insomnia reaches up to US $100 billion [9]. In addition, insomnia contributes to substantial losses in annual gross domestic product, amounting to US $19.6 billion in Canada, US $41.4 billion in the United Kingdom, and US $19.2 billion in Australia [10]. The cost of untreated insomnia outweighs the cost of treating insomnia, with cognitive behavioral therapy for insomnia (CBTi) achieving greater cost-effectiveness than pharmacological treatments [11]. Technology-aided delivery of CBTi may have even greater cost-effectiveness, given its high scalability and reduced demands for human resources compared to face-to-face CBTi [12].

CBTi is an evidence-based, first-line treatment for insomnia disorder recommended by health organizations around the world [13,14]. It targets the cognitive and behavioral mechanisms perpetuating sleep difficulties, namely compromised sleep drive, disturbed circadian rhythm, and hyperarousal, especially hyperarousal associated with the sleeping environment or sleep per se [15-17]. Integrating multiple treatment techniques, CBTi aims to preserve sleep drive, stabilize circadian rhythm, and reduce hyperarousal; it is typically delivered in 6 to 8 hourly sessions by a trained mental health professional [18]. CBTi effectively improves sleep quality and reduces insomnia symptoms across populations, including populations with medical and psychiatric comorbidities. On average, CBTi leads to 20- to 30-minute reductions in both sleep onset latency and wake after sleep onset and approximately 10% increases in sleep efficiency at the end of treatment [19] with less consistent effects on total sleep time (TST) [20]. CBTi also improves mood, fatigue, and quality of life—indirectly through sleep improvements or directly through changes in behavior and cognition [21,22]. Despite its strong evidence base, CBTi is not consistently delivered to most people due to various implementation obstacles, notably the lack of mental health workers trained in delivering CBTi and limitations in time and resources required for delivering and receiving CBTi [23].

Digital CBTi (dCBTi) is a promising alternative mode of delivery of CBTi, given its high scalability and low demands for human resources. Meta-analyses have found comparable efficacy estimates of dCBTi compared to face-to-face CBTi [24,25]. However, a direct comparative trial found dCBTi less efficacious than face-to-face CBTi [26]. Notably, fully automated dCBTi is the most cost-effective treatment of insomnia, followed by group CBTi and individual CBTi [27]. Of the different types of dCBTi, dCBTi with therapist support is found to have the highest efficacy compared to other types of dCBTi without therapist support [28]. Indeed, CBTi recipients attribute treatment success to the working alliance with the therapist, and they perceive therapist-assisted support, such as the provision of personalized feedback, motivational enhancement, and accountability, to be critical for increasing engagement with dCBTi [23]. Nonetheless, the need for therapist support hinders the scalability and accessibility of dCBTi, which are the core benefits of dCBTi over face-to-face CBTi.

dCBTi with nontherapist guidance is a lower-cost alternative. Although it may be limited in delivering expert advice and addressing challenging motivational or emotional barriers, nontherapist support can provide personalized feedback, motivational enhancement, and accountability. Nontherapist support has been found to improve treatment outcomes of self-help CBTi [29]. Technological advancements have enabled the development of virtual conversational agents designed to mimic patient-therapist interactions and provide personalized content and feedback, also known as chatbot-based coaching or e-coaching [30]. Nontherapist and chatbot-based support require no therapist and preserve the core benefit of dCBTi over face-to-face CBTi. Meta-analyses have found mixed results regarding the effect sizes of dCBTi with different types of coaching support [24,25], but a direct comparative trial has not yet been conducted.

Low treatment adherence and high attrition rates are the major challenges in implementing dCBTi and even face-to-face CBTi [31,32]. On average, half of the dCBTi recipients do not adhere to treatment [33] compared to 14% to 44% in face-to-face CBTi [34]. While dCBTi offers greater potential for scalability, if engagement and treatment adherence are low, its impact on population health will remain minimal. Adding human or chatbot-based guidance to dCBTi is one of the most discussed solutions to improving engagement [31,33]; however, empirical support for its effects on treatment adherence is still being determined.

In sum, dCBTi is undoubtedly a promising intervention for insomnia that could have a major impact at the population level. Nevertheless, more research on how to optimize its benefits is needed. In particular, adding human or chatbot-based coaching has been frequently regarded as a useful strategy to improve treatment efficacy. However, a direct comparative trial of dCBTi with different types of coaching compared to dCBTi without any coaching (dCBTi-unguided) has yet to be conducted. Furthermore, the effects of different types of coaching on treatment adherence have not been evaluated.

### Objectives

This study is the first empirical evaluation of the efficacy of dCBTi with different types of coaching. Moreover, we aim to evaluate whether different coaching types improve treatment adherence along with treatment outcomes. Specifically, in a 5-arm randomized controlled comparative trial, we aim to evaluate whether (1) a fully automated mobile phone–based dCBTi has superior efficacy to an active digital sleep hygiene and self-monitoring control (dSH); (2) adding coaching, regardless of type, would enhance treatment adherence and efficacy compared to dCBTi-unguided; (3) dCBTi with human support would enhance treatment adherence and efficacy compared to dCBTi with chatbot-based coaching only (dCBTi-chatbot); and (4) support from a therapist is superior to support from a nontherapist. We hypothesized that (1) dCBTi, regardless of the presence of coaching, would be efficacious for improving insomnia symptoms compared to dSH; (2) dCBTi with coaching would promote greater improvements in insomnia symptoms and greater treatment adherence than dCBTi-unguided; (3) dCBTi with human coaching would promote greater improvements in insomnia symptoms and treatment adherence than dCBTi-chatbot; and (4) dCBTi with chatbot-based coaching and therapist support (dCBTi-therapist) would promote greater improvements in insomnia symptoms and greater treatment adherence than dCBTi with chatbot-based coaching and research assistant support (dCBTi-assistant).

## Methods

### Study Design

The study was a 5-arm, parallel, participant-blinded, randomized controlled comparative trial. The 5 conditions included dCBTi-therapist, dCBTi-assistant, dCBTi-chatbot, dCBTi-unguided, and dSH. The study protocol was preregistered on ClinicalTrials.gov (NCT05136638).

### Ethical Considerations

This study was approved by the University of Hong Kong Human Research Ethics Committee before data collection (EA210458). Electronic informed consent was obtained from each participant before study participation. Each participant was informed that participation was entirely voluntary, and they could withdraw from the study at any point without negative consequences. All data were kept confidential in a password-protected drive. Only the research team had access to the data. All personal identifying information was removed from the research data and will be kept separately from the research data for 3 years after the publication of the main study findings to ensure that there are no problems regarding consent, fabrication, or falsification. Anonymous data will be kept indefinitely. Each participant was compensated HK $60 (approximately US $8) for completing research measures at each time point.

### Randomization and Blinding

Simple randomization with equal chances of assigning a participant to 1 of the 5 conditions was conducted using the randomization function implemented in Sleep Sensei, a mobile app developed with the MobileCoach platform [35,36] specifically for this study. Participants were informed that they were assigned to one of the intervention conditions. However, they were not informed about the conditions or the condition to which they were assigned. They were not informed about the study hypotheses either. As all participants were given access to Sleep Sensei, we considered them blinded to the condition assignment and study hypotheses. The therapists and assistants who provided coaching support were not blinded to the assignment. The assessments of treatment outcomes were all self-administered using Qualtrics (Qualtrics International Inc).

### Participants

The inclusion criteria were as follows: participants who (1) have an Insomnia Severity Index (ISI) score of ≥10, indicating clinically significant insomnia [37]; (2) are aged 18 to 65 years; (3) have access to a smartphone and a local telephone number; and (4) are able to understand written Chinese and spoken Cantonese, the languages used in Sleep Sensei. The exclusion criteria were as follows: (1) self-reported sleep apnea or high risk of sleep apnea identified using the Berlin Questionnaire [38]; (2) self-reported acute, untreated mental or medical illnesses that would interfere with participation; (3) suicidal ideation suggested by a score of ≥1 on the Patient Health Questionnaire-9 (PHQ-9) and confirmed by a follow-up interview by a clinical psychology trainee; (4) unstable medication use that can affect sleep; (5) currently receiving psychotherapy for insomnia; and (6) other conditions that prevent adherence to CBTi recommendations, such as shift work. Participants who did not complete baseline research assessments were also excluded. Eligible participants showed sufficient digital literacy to be able to complete the web-based screening survey and use the mobile app.

An a priori power analysis was conducted to determine the sample size required for detecting significant group-by-time interaction effects if there were clinically meaningful differences in the primary outcome (a 4-point difference in the ISI total score [39]) in the patterns specified in our 4 hypotheses. A simulation-based power analysis was performed using the R package *mixedpower* [40]. We simulated a database using the means and SDs of ISI scores from a local sample for another insomnia trial as the baseline values [41] because these data were most likely the closest estimates of baseline ISI values in our study sample. For hypothesis 1, we simulated a database with after-treatment ISI values to be 4 points lower than the baseline ISI values in the experimental condition. For hypotheses 2 to 4, we simulated a database with after-treatment ISI values to be 4 points lower than the baseline's and 4 points lower than the comparison group's. On the basis of these simulations, a sample size of 120 would be required for detecting significant results with statistical power >95%, >80%, >90%, and >80% for hypotheses 1, 2, 3, and 4, respectively.

### Procedures

**Figure 1:**
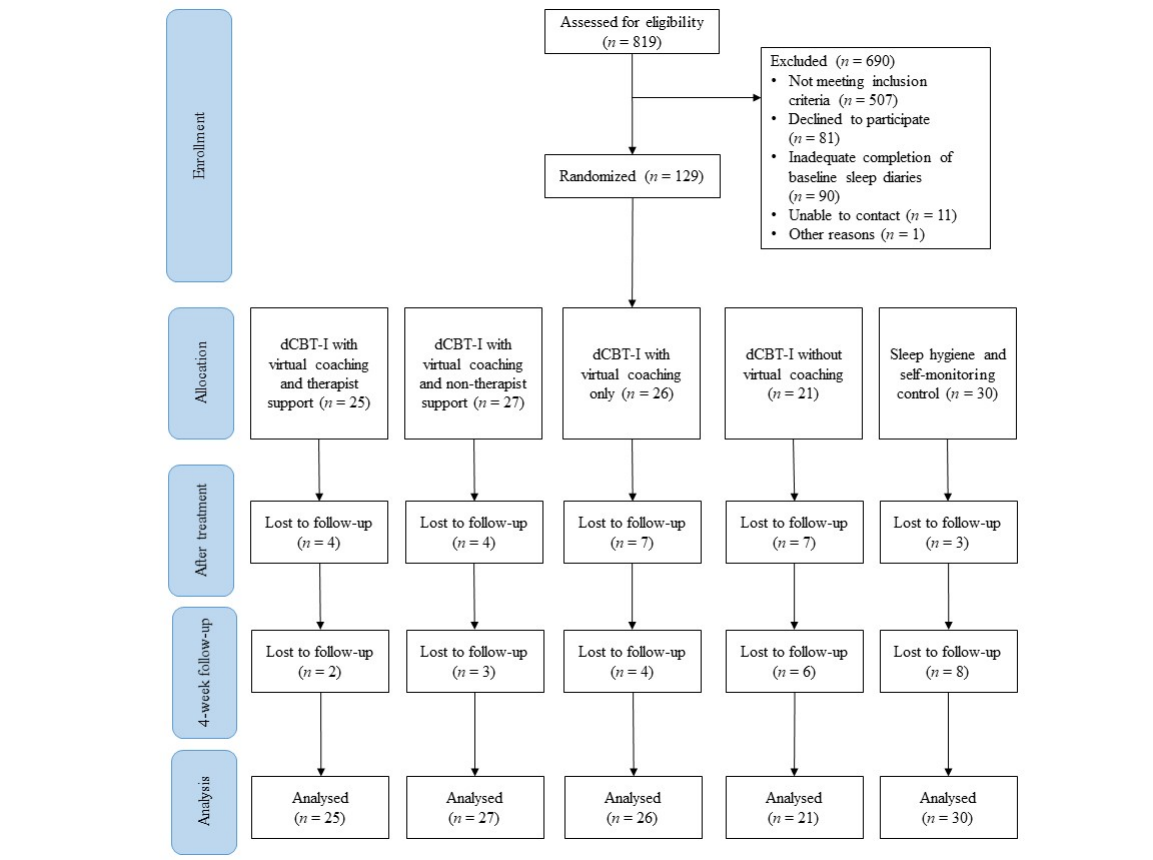
CONSORT (Consolidated Standards of Reporting Trials) flowchart of participants. dCBTi: digital cognitive behavioral therapy for insomnia.

Participants were recruited using mass emails sent to students, staff, and affiliates of the university, as well as social media advertisements with the institutional affiliation displayed. Inclusion and exclusion criteria were evaluated based on potential participants’ responses to the screening survey, followed by telephone interviews as needed by authors SHCL and AKMC, both of whom were clinical psychology trainees under the supervision of lead author WSC, a licensed clinical psychologist. Eligible participants then downloaded Sleep Sensei for free. All conditions were delivered via Sleep Sensei. They were given access to modules and functions according to the condition to which they were assigned (Table 1). They had access to Sleep Sensei from baseline to follow-up. Assessments of outcomes were administered at baseline, after treatment, and at 4-week after-treatment (follow-up) using Qualtrics. Figure 1 presents the CONSORT (Consolidated Standards of Reporting Trials) flow diagram.

**Table 1:** Intervention components in each treatment condition.

Treatment components	dCBTi-therapist^a^	dCBTi-assistant^b^	dCBTi-chatbot^c^	dCBTi-unguided^d^	dSH^e^
Video lessons	✓	✓	✓	✓	✓^f^
Resource library	✓	✓	✓	✓	✓^f^
Daily diary entry and visualization	✓	✓	✓	✓	✓
Automatic customized sleep schedule suggestions	✓	✓	✓	✓	
Weekly goal-setting and action-planning entries	✓	✓	✓	✓	
Chatbot-based coaching	✓	✓	✓		
Assistant support		✓			
Therapist support	✓				

^a^dCBTi-therapist: digital cognitive behavioral therapy for insomnia with chatbot-based coaching and therapist support.

^b^dCBTi-assistant: digital cognitive behavioral therapy for insomnia with chatbot-based coaching and research assistant support.

^c^dCBTi-chatbot: digital cognitive behavioral therapy for insomnia with chatbot-based coaching only.

^d^dCBTi-unguided: digital cognitive behavioral therapy for insomnia without any coaching.

^e^dSH: digital sleep hygiene and self-monitoring control.

^f^Sleep hygiene only.

### Interventions

#### Sleep Sensei

Sleep Sensei was developed specifically for this study and hence was customized to deliver the 5 conditions with differing combinations of content and functionalities. Sleep Sensei used the talk-and-tools user-interface paradigm, which comprised a *talk* system enabling text-based chat interactions between the user and a conversational agent (ie, a chatbot-based coach) and a *tools* system allowing the user to observe and manipulate the objects in the interface [30]. Before launching the app, Sleep Sensei underwent 3 rounds of usability testing with 6 volunteers.

In Sleep Sensei, the core CBTi treatment components were implemented using the tools system, which consisted of (1) 6 video lessons providing psychoeducation and the rationale for each treatment recommendation, delivered chronologically in the order described in the next subsection; (2) a resource library storing and presenting video lesson content in written format as well as additional resources such as relaxation recordings; (3) daily diary entries and visualization of diary data; (4) automatic, individually tailored weekly sleep schedule suggestions; and (5) weekly goal-setting and action-planning entries. Multimedia Appendix 1 presents screenshots of the Sleep Sensei interface.

#### CBTi Core Treatment Components

CBTi is a multicomponent intervention that combines behavioral techniques and cognitive therapy [14]. The core treatment components of CBTi include sleep restriction, stimulus control, sleep hygiene, psychoeducation about sleep, relaxation, and cognitive therapy. Sleep hygiene is included in CBTi but is not considered CBTi on its own. It is often used as an active control condition in clinical trials of CBTi [42].

Sleep restriction is a technique to improve one’s sleep efficiency by restoring a high sleep drive via limiting time in bed (TIB), specifically to match one’s current sleep needs. It is achieved by tailoring TIB to match one’s average TST. Once adequate sleep efficiency is achieved, TIB can gradually extend until optimal sleep duration is reached. In this study, the rationale and procedures for sleep restriction are presented in video lesson 1. If the participant’s previous-week sleep diary data show that their sleep efficiency is ≥85%, and they indicated that they were not sleep deprived by answering a *yes* or *no* question, they would be asked to maintain their TIB with consistent wake time and bedtime. If their sleep efficiency is ≥85%, and they indicated that they were sleep deprived, the app would recommend TIB with a 20-minute increase. If their sleep efficiency is <85%, the app would recommend a range of TIBs, from the minimum TST (equivalent to the previous week’s average TST based on diary data but at least 5 hours) to 20 minutes less than the previous week’s TIB. The participant will be asked to specify a consistent wake time suitable for them and choose a bedtime that would result in a TIB within the suggested range. This implementation of the sleep restriction procedures enables the participant to choose to restrict TIB more aggressively or gradually by 20 minutes each week, allowing for greater flexibility and potentially greater adherence.

Sleep hygiene refers to daily habits that influence sleep drive, circadian rhythm, or arousal associated with the sleeping environment. The daily habits include keeping the sleeping environment dark, quiet, and cool; having a consistent sleep schedule, especially a consistent wake time; maintaining adequate light exposure and activity levels; reducing stimulating activities before bedtime such as eating too much, intense exercise, alcohol or nicotine consumption, and screen time; and setting up a relaxation routine before bedtime. The rationale and procedures for sleep hygiene are presented in video lesson 2.

Stimulus control refers to reconditioning the sleeping environment to be a place only for sleeping and to reduce conditioned arousal. The procedures instruct the participant to enter the sleeping environment only when they feel ready to fall asleep, leave the sleeping environment if they cannot fall asleep or stay asleep for approximately >20 minutes by estimation, and go back to bed only when one feels sleepy. The distinction between feeling fatigued and sleepy is also discussed. The rationale and procedures for stimulus control are presented in video lesson 3.

Relaxation includes practices of progressive muscle relaxation and visual imagery exercises that can be used to reduce hyperarousal. The rationale and procedures for relaxation are presented in video lesson 4. Participants are guided to practice relaxation during the video lesson and told that recordings of relaxation guidance could be accessed in the resource library. In addition, another clinical technique targeting excessive worry is also introduced in this lesson—setting up worry time (referring to setting aside a 30-minute period each day [not close to bedtime] for worrying) and limiting worrying only to this time to restrict the impact of worrying on mental health and sleep.

Cognitive therapy refers to a set of techniques to identify and reframe thoughts and beliefs that may promote and sustain sleep difficulties, such as thoughts that lead to heightened worries and frustration about the consequences of not having good sleep or thoughts that reduce motivation and treatment adherence. The rationale and procedures for cognitive therapy are presented in video lessons 5 and 6.

#### Chatbot-Based Coaching

The chatbot-based coaching element was delivered using the *talk* system via a series of logic-based preprogrammed conversational turns created by WSC, SHCL, and AKMC with the goal to simulate therapeutic interactions. These text-based conversations covered the following areas: (1) after-video summary and quiz (the chatbot-based coach guided the participants to reflect on what they learned from the video lessons and facilitated them to apply the learned strategies to their own situations), (2) weekly goal setting and action planning (the participants were guided to set up goals and action plans to implement the treatment recommendations during the week), (3) positive feedback and reflection (encouraging messages were sent to participants when they completed daily sleep diaries and achieved their weekly goals), and (4) problem-solving (when the participants did not complete daily diaries or achieve weekly goals, the chatbot-based coach guided them to think about different solutions to remove the barriers to implement the treatment recommendations).

#### Research Assistant Support

Research assistant support was provided to participants in dCBTi-assistant at the end of sessions 1, 3, and 6 by 2 undergraduate research interns who had no prior experience in counseling or CBTi. They were trained by WSC to provide supportive contact, including expressing appreciation for the participants’ time and effort in using the app, encouraging them to continue using the app and complete daily diaries, and asking whether they had encountered any technical issues that needed support. If the participant asked sleep- or CBTi-related questions, the assistants would direct the participant to review the CBTi materials on the app. The contact time was restricted to between 20 and 30 minutes. The percentages of telephone calls completed were 74% (20/27), 33% (9/27), and 30% (8/27) for sessions 1, 3, and 6, respectively.

#### Therapist Support

Therapist support was provided to participants in dCBTi-therapist at the end of sessions 1, 3, and 6 by 2 postgraduate clinical psychology trainees (SHCL and AKMC) who had received at least 1 year of clinical psychology training and training in a CBTi protocol. The support included reinforcing the understanding of the intervention materials, providing sleep- and CBTi-related expert advice, reviewing treatment progress, identifying and resolving barriers to implementing CBTi treatment strategies, and addressing any motivational issues. Similar to the dCBTi-assistant condition, the contact time was restricted to between 20 and 30 minutes. The percentages of telephone calls completed were 84% (21/25), 76% (19/25), and 68% (17/25) for sessions 1, 3, and 6, respectively.

### Measures

#### Overview

The assessments were all electronic questionnaires administered using Qualtrics. These questionnaires had been tested by research assistants before being distributed to the participants. The questionnaires were distributed through email, and participants were instructed to complete them within 1 week. The order of the questions was the same for each participant. All questions were mandatory.

#### Primary Outcome (ISI)

The ISI is a widely used self-report questionnaire assessing insomnia symptoms in the previous 2 weeks [43]. It consists of 7 items asking the participants to rate the severity of their insomnia symptoms on a 5-point Likert scale ranging from 0 to 4, with higher scores indicating greater insomnia severity. The composite score ranges from 0 to 28, with a score of ≥10 indicating clinical insomnia. The validated Chinese version was used in this study [44]. The ISI showed acceptable to excellent internal consistencies across the 3 time points, with Cronbach α values in the range of 0.77 to 0.90.

#### Secondary Outcomes

##### Sleep Condition Indicator

The Sleep Condition Indicator (SCI) is a newer measure of insomnia symptoms developed based on the *Diagnostic and Statistical Manual of Mental Disorders, Fifth Edition* (*DSM-5*), diagnostic criteria [1]; research diagnostic criteria [45]; and recommended quantitative parameters [46]. It consists of 8 items assessing the severity of sleep difficulties and daytime impairment during the past month. Four items assess insomnia symptoms on 5-point scales with quantitative anchors (ie, frequency or duration); for instance, item 1 asks, “How long does it take for you to fall asleep?” and the participants respond on a scale ranging from 0 to 4 (0=*0-15 min*, 1=*16-30 min*, 2=*31-45 min*, 3=*46-60 min*, and 4=*>61 min*). The other 4 items assess insomnia symptoms on 5-point scales with qualitative anchors; for instance, item 4 asks, “How do you rate your sleep quality?” and the participants respond on a scale ranging from 0 (*very good*) to 4 (*very bad*). The SCI has been validated against the ISI, and it has shown good reliability and convergent validity. The validated Chinese version of the SCI was used in this study [47]. The total score ranges from 0 to 32; higher scores indicate lower levels of insomnia, and a score of ≤16 indicates insomnia disorder. In this study, the SCI had acceptable to good internal consistency, with Cronbach α values in the range of 0.70 to 0.88.

##### PHQ-9 for Depressive Symptoms

The PHQ-9 was used to assess depressive symptoms [48]. The PHQ-9 consists of 9 items asking participants to rate the frequency of their depressive symptoms in the past 2 weeks on a 4-point Likert scale ranging from 0 (*not at all*) to 3 (*nearly every day*). The total score ranges from 0 to 27; higher scores indicate higher levels of depressive symptoms, with a PHQ-9 cutoff score of ≥10 indicating clinically significant depressive symptoms. The validated Chinese version was used in this study [49]. The PHQ-9 showed good internal consistencies across the 3 time points, with Cronbach α values in the range of 0.81 to 0.88.

##### Generalized Anxiety Disorder-7

The Generalized Anxiety Disorder-7 (GAD-7) was used to measure anxiety symptoms [50]. The GAD-7 consists of 7 items asking participants to rate the frequency of their anxiety symptoms in the past 2 weeks on a 4-point Likert scale ranging from 0 (*not at all*) to 3 (*nearly every day*)*.* The composite score ranges from 0 to 21; higher scores indicate higher levels of anxiety symptoms, with a GAD-7 cutoff score of ≥8 indicating clinically significant anxiety symptoms. The validated Chinese version was used in this study [51]. The GAD-7 showed excellent internal consistencies across the 3 time points, with Cronbach α values in the range of 0.92 to 0.95.

##### Fatigue Assessment Scale

The Fatigue Assessment Scale (FAS) was used to assess fatigue [52]. The FAS consists of 10 items asking participants to rate the frequency of their fatigue symptoms on a 5-point Likert scale ranging from 1 (*never*) to 5 (*always*). The total score ranges from 10 to 50, with higher scores indicating higher levels of fatigue. The validated Chinese version was used in this study [53]. In this study, the FAS had good internal consistencies across the 3 time points, with Cronbach α values in the range of 0.88 to 0.90.

##### Epworth Sleepiness Scale

The validated Chinese version of the Epworth Sleepiness Scale (ESS) was used to assess daytime sleepiness [54]. The ESS consists of 8 items asking participants to rate their chances of dozing in different situations on a 4-point Likert scale, ranging from 0 (*never*) to 3 (*high chance*)*.* The composite score ranges from 0 to 24, and higher scores indicate greater daytime sleepiness, with an ESS score of ≥11 indicating excessive or clinically significant daytime sleepiness. The ESS showed good internal consistencies across the 3 time points, with Cronbach α values in the range of 0.82 to 0.83.

##### Satisfaction With Life Scale

The Satisfaction With Life Scale (SWLS) was used to measure general psychological well-being [55]. The SWLS asks participants to rate their agreement with 5 statements of life satisfaction on a 7-point Likert scale ranging from 1 (*strongly disagree*) to 7 (*strongly agree*)*.* The total score ranges from 5 to 35; higher scores indicate greater psychological well-being. The validated Chinese version was used in this study [56]. The SWLS showed good internal consistencies across the 3 time points, with Cronbach α values in the range of 0.90 to 0.92.

#### Mechanistic Outcomes

##### Dysfunctional Beliefs and Attitudes About Sleep-16

The Dysfunctional Beliefs and Attitudes About Sleep-16 (DBAS-16) measures one’s endorsement of dysfunctional thoughts and beliefs that could elevate anxiety and frustration about sleep difficulties, leading to the perpetuation of insomnia [57]; for example, 1 of the items is “I need 8 hours of sleep to feel refreshed and function well during the day.” The DBAS-16 asks participants to rate how much they believe the 16 statements about sleep on an 11-point Likert scale ranging from 0 (*strongly disagree*) to 10 (*strongly agree*). The total score ranges from 0 to 160; higher scores indicate stronger dysfunctional beliefs about sleep. The validated Chinese version was used in this study [58]. The DBAS-16 showed good to excellent internal consistencies across the 3 time points, with Cronbach α values in the range of 0.85 to 0.95.

##### Sleep-Related Behaviors Questionnaire

The Sleep-Related Behaviors Questionnaire (SRBQ) measures one’s engagement in sleep-related safety behaviors, which are behaviors that aim to alleviate the distress and consequences of insomnia but inadvertently perpetuate insomnia [59] (eg, preoccupation with sleep, such as “I spend time considering ways to improve sleep”; and reduced daytime engagement to preserve energy, such as “I take on fewer social commitments”). The validated Chinese version of the SRBQ, which consists of 20 statements, was used [41]. Participants rated these statements on a 5-point Likert scale ranging from 0 (*almost never*) to 4 (*almost always*). The composite score ranges from 0 to 80; higher scores indicate greater engagement in sleep-related safety behaviors. The SRBQ had good to excellent internal consistencies across the 3 time points, with Cronbach α values in the range of 0.85 to 0.93.

#### Treatment Adherence

In this study, video completion and sleep diary completion for each dCBTi session were used as the indicators of treatment adherence, which are common global indicators of treatment adherence that have been used in other CBTi and dCBTi trials [60].

### Statistical Analysis

Analyses were performed with R (version 4.0.2; R Foundation for Statistical Computing). All tests for significance were 2-tailed, and *P*<.05 was considered statistically significant. Intention-to-treat analyses were conducted using linear mixed models with the restricted maximum likelihood method for handling missing data. The restricted maximum likelihood method incorporates the observed data and model covariance structure to estimate the variance parameters in the model with missing data [61]. The models included the treatment groups (dCBTi-therapist, dCBTi-assistant, dCBTi-chatbot, dCBTi-unguided, and dSH), time (baseline, after treatment, and follow-up), and the group-by-time interaction as fixed effects. Participant IDs were included as the random effect in the model. Planned contrasts were specified in the models to test our 4 hypotheses: hypothesis 1, all dCBTi conditions compared to dSH; hypothesis 2, all guided dCBTi conditions compared to unguided dCBTi; hypothesis 3, dCBTi with human support compared to dCBTi-chatbot; and hypothesis 4, dCBTi-therapist compared to dCBTi-assistant.

Cohen *d* was calculated from the mean differences between the conditions after treatment to indicate the effect size of each significant effect. Fisher exact tests were conducted to evaluate whether the percentages of participants who completed the video and sleep diary for each session were different across the conditions. In addition, we conducted chi-square tests on remission rates to evaluate the differences in remission rates across the conditions after treatment. Remission was defined as having an ISI score of <10 or an SCI score of >21. We also conducted an analysis on the percentages of participants achieving a clinically meaningful reduction in depressive symptoms (a 5-point reduction in the PHQ-9 score), anxiety symptoms (a 4-point reduction in the GAD-7 score), fatigue (a 4-point reduction in the FAS score), and daytime sleepiness (a 2-point reduction in the ESS score).

## Results

### Descriptives

Of the 819 individuals who completed the screening survey, 690 (84.2%) were not eligible, declined to participate, or did not complete the baseline measures; thus the final sample consisted of 129 (15.8%) participants (age: mean 34.09, SD 12.05 y). Most of the participants were female (98/129, 76%), had never married (87/129, 67.4%), had completed tertiary education (104/129, 80.6%), and were employed full time (77/129, 59.7%). There were no significant differences in age, marital status, education level, employment status, or monthly household income across treatment conditions (Table 2). Table 3 presents the mean values and SDs of each outcome at 3 time points. No significant differences were observed in all outcomes at baseline. Significant differences in the ISI, SCI, PHQ-9, GAD-7, DBAS-16, and SRBQ scores across the conditions were observed after treatment, favoring the treatment conditions over the control.

**Table 2:** Demographic characteristics of participants at baseline.

Characteristics			dCBTi-therapist^a^ (n=25)		dCBTi-assistant^b^ (n=27)		dCBTi-chatbot^c^ (n=26)		dCBTi-unguided^d^ (n=21)		dSH^e^ (n=30)		Full sample (n=129)		*F* test (*df*)			Chi-square (*df*)			*P* value	
Age (y), mean (SD)			34.28 (12.18)		34.22 (13.62)		35.58 (12.23)		30.76 (10.45)		34.83 (11.68)		34.09 (12.05)		0.52 (4,124)			—^f^			.72	
**Sex, n (%)**																—			10.2 (4)			.04
	Female	18 (72)		21 (77.8)		21 (80.8)		11 (52.4)		27 (90)		98 (76)										
	Male	7 (28)		6 (22.2)		5 (19.2)		10 (47.6)		3 (10)		31 (24)										
**Marital status, n (%)**																—			17.1 (12)			.15
	Never married	16 (64)		20 (74.1)		14 (53.8)		18 (85.7)		19 (63.3)		87 (67.4)										
	Cohabiting	2 (8)		0 (0)		3 (11.5)		0 (0)		0 (0)		5 (3.9)										
	Married	5 (20)		5 (18.5)		9 (34.6)		2 (9.5)		10 (33.3)		31 (24)										
	Divorced or separated	2 (8)		1 (7.4)		0 (0)		1 (4.8)		1 (3.3)		6 (4.7)										
**Highest educational level, n (%)**																—			12.1 (8)			.15
	Secondary	4 (16)		2 (7.4)		4 (15.4)		0 (0)		2 (6.7)		12 (9.3)										
	Tertiary (nondegree)	3 (12)		3 (11.1)		1 (3.8)		0 (0)		6 (20)		13 (10.1)										
	Tertiary (degree)	18 (72)		22 (81.5)		21 (80.8)		21 (100)		22 (73.3)		104 (80.6)										
**Employment status, n (%)**																—			13.9 (20)			.84
	Full time	15 (60)		15 (55.6)		19 (73.1)		13 (61.9)		15 (50)		77 (59.7)										
	Part time	1 (4)		3 (11.1)		0 (0)		1 (4.8)		4 (13.3)		9 (7)										
	Unemployed	1 (4)		2 (7.4)		1 (3.8)		1 (4.8)		2 (6.7)		7 (5.4)										
	Retired	1 (4)		1 (3.7)		2 (7.7)		0 (0)		1 (3.3)		5 (3.9)										
	Homemaker	1 (4)		0 (0)		0 (0)		0 (0)		2 (6.7)		3 (2.3)										
	Student	6 (24)		6 (22.2)		4 (15.4)		6 (28.6)		6 (20)		28 (21.7)										
**Monthly household income, HK $ (US $), n (%)**																—			12.3 (20)			.71
	<15,000 (1950)	7 (28)		9 (33.3)		4 (15.4)		4 (19)		9 (30)		33 (25.6)										
	15,000 (1950)-24,999 (3249.87)	5 (20)		4 (14.8)		4 (15.4)		6 (28.6)		7 (23.3)		26 (20.2)										
	25,000 (3250)-39,999 (5199.87)	6 (24)		6 (22.2)		11 (42.3)		5 (23.8)		5 (16.7)		33 (25.6)										
	40,000 (5200)-59,999 (7799.87)	3 (12)		4 (14.8)		3 (11.5)		2 (9.5)		1 (3.3)		13 (10.1)										
	>60,000 (7800)	4 (16)		4 (14.8)		2 (7.7)		4 (19)		7 (23.3)		21 (16.28)										

^a^dCBTi-therapist: digital cognitive behavioral therapy for insomnia with chatbot-based coaching and therapist support.

^b^dCBTi-assistant: digital cognitive behavioral therapy for insomnia with chatbot-based coaching and research assistant support.

^c^dCBTi-chatbot: digital cognitive behavioral therapy for insomnia with chatbot-based coaching only.

^d^dCBTi-unguided: digital cognitive behavioral therapy for insomnia without any coaching.

^e^dSH: digital sleep hygiene and self-monitoring control.

^f^Not applicable.

**Table 3:** Measures of outcomes by time points.

Variables and time points		dCBTi-therapist^a^ (n=25), mean (SD)	dCBTi-assistant^b^ (n=27), mean (SD)	dCBTi-chatbot^c^ (n=26), mean (SD)	dCBTi-unguided^d^ (n=21), mean (SD)	dSH^e^ (n=30), mean (SD)	Full sample (n=129), mean (SD)	*F* test (*df*)	*P* value
**ISI^f^**									
	Baseline	14.96 (3.65)	13.70 (3.86)	14.50 (4.96)	15.76 (5.17)	16.30 (4.39)	15.05 (4.44)	1.47 (4,124)	.22
	After treatment	9.10 (4.23)^g^	9.00 (4.59)^g^	9.42 (4.44)	9.64 (5.00)	13.22 (5.07)^g^	10.28 (4.92)	3.66 (4,99)	.01
	Follow-up	9.26 (4.85)	7.55 (4.86)	10.00 (5.32)	6.88 (4.67)	12.06 (6.20)	9.36 (5.43)	2.28 (4,75)	.07
**SCI^h^**									
	Baseline	11.88 (4.22)	13.48 (5.28)	12.81 (5.21)	12.81 (5.01)	11.70 (4.62)	12.51 (4.85)	0.62 (4,124)	.65
	After treatment	19.86 (4.90)^i^	18.30 (5.80)	19.16 (6.48)^i^	19.43 (7.55)	13.96 (6.03)^i^	17.80 (6.41)	3.81 (4,99)	.01
	Follow-up	21.00 (6.38)	20.40 (7.00)	19.00 (6.11)	22.75 (4.71)	15.67 (8.13)	19.45 (7.00)	2.23 (4,75)	.07
**PHQ-9^j^**									
	Baseline	11.60 (5.32)	9.52 (4.59)	10.16 (4.61)	12.86 (6.33)	10.38 (5.36)	10.80 (5.28)	1.50 (4,122)	.21
	After treatment	7.10 (5.35)	6.35 (5.02)^k^	7.16 (3.86)	8.07 (5.41)	10.92 (6.36)^k^	8.04 (5.51)	2.77 (4,98)	.03
	Follow-up	6.74 (5.43)	6.40 (5.50)	7.47 (4.66)	7.86 (4.74)	9.44 (6.24)	7.51 (5.44)	0.87 (4,74)	.48
**GAD-7^l^**									
	Baseline	9.52 (5.55)	7.44 (5.06)	8.92 (4.56)	11.95 (5.67)	9.69 (5.86)	9.40 (5.46)	2.16 (4,122)	.08
	After treatment	6.10 (3.99)	5.35 (5.01)^m^	6.05 (4.56)	8.79 (6.28)	9.54 (6.04)^m^	7.16 (5.40)	2.82 (4,98)	.03
	Follow-up	7.42 (6.09)	6.05 (4.95)	6.87 (5.25)	9.43 (7.50)	7.33 (5.98)	7.13 (5.69)	0.48 (4,74)	.75
**FAS^n^**									
	Baseline	32.24 (7.23)	28.74 (7.07)	28.04 (7.20)	30.81 (7.40)	31.00 (7.61)	30.15 (7.36)	1.42 (4,122)	.23
	After treatment	25.81 (6.27)	25.61 (5.61)	26.74 (8.09)	27.64 (8.58)	30.54 (8.52)	27.38 (7.56)	1.77 (4,98)	.14
	Follow-up	25.47 (8.20)	25.75 (6.89)	27.87 (7.37)	27.57 (7.02)	28.56 (6.96)	26.89 (7.27)	0.61 (4,74)	.66
**ESS^o^**									
	Baseline	9.32 (4.63)	10.59 (4.76)	10.24 (4.38)	11.86 (4.61)	8.55 (5.10)	10.12 (4.77)	1.75 (4,122)	.14
	After treatment	8.57 (4.51)	8.57 (4.53)	10.47 (4.23)	9.36 (5.68)	8.23 (5.22)	8.94 (4.80)	0.71 (4,98)	.59
	Follow-up	7.16 (5.21)	8.30 (4.43)	10.67 (3.70)	11.29 (5.94)	7.33 (4.43)	8.52 (4.78)	2.13 (4,74)	.08
**SWLS^p^**									
	Baseline	14.64 (6.25)	17.26 (6.17)	18.00 (6.43)	13.48 (5.20)	17.34 (5.25)	16.28 (6.04)	2.63 (4,122)	.04
	After treatment	18.29 (7.24)	19.74 (6.05)	18.00 (6.72)	15.86 (6.31)	17.54 (5.46)	18.04 (6.32)	0.88 (4,98)	.48
	Follow-up	19.16 (8.39)	20.00 (5.67)	19.47 (6.15)	19.43 (6.58)	18.17 (5.76)	19.23 (6.47)	0.19 (4,74)	.94
**DBAS-16^q^**									
	Baseline	6.22 (1.23)	5.94 (1.71)	6.09 (1.20)	6.04 (1.18)	6.20 (1.68)	6.10 (1.42)	0.17 (4,124)	.95
	After treatment	2.51 (2.27)^r^	3.33 (2.14)^r^	3.50 (2.57)^r^	2.73 (2.54)^r^	5.55 (2.74)^r^	3.63 (2.68)	6.67 (4,124)	<.001
	Follow-up	2.55 (2.53)	2.57 (2.43)	2.99 (2.81)	1.87 (2.77)	3.30 (3.02)	2.71 (2.73)	0.96 (4,124)	.43
**SRBQ^s^**									
	Baseline	37.68 (9.21)	32.85 (11.50)	36.20 (9.46)	34.43 (10.40)	36.47 (10.30)	35.55 (10.21)	0.89 (4,123)	.47
	After treatment	28.05 (11.11)	27.13 (10.15)^t^	29.11 (11.36)	30.57 (14.66)	38.73 (15.62)^t^	31.08 (13.32)	3.30 (4,98)	.01
	Follow-up	24.11 (14.36)	25.00 (11.97)	30.53 (11.72)	29.38 (11.71)	34.44 (13.69)	28.39 (13.22)	1.97 (4,75)	.11

^a^dCBTi-therapist: digital cognitive behavioral therapy for insomnia with chatbot-based coaching and therapist support.

^b^dCBTi-assistant: digital cognitive behavioral therapy for insomnia with chatbot-based coaching and research assistant support.

^c^dCBTi-chatbot: digital cognitive behavioral therapy for insomnia with chatbot-based coaching only.

^d^dCBTi-unguided: digital cognitive behavioral therapy for insomnia without any coaching.

^e^dSH: digital sleep hygiene and self-monitoring control.

^f^ISI: Insomnia Severity Index.

^g^Significant differences between treatment groups in post hoc multiple comparisons (dCBTi-therapist vs dSH, *P*=.03; dCBTi-assistant vs dSH, *P*=.02).

^h^SCI: Sleep Condition Indicator.

^i^Significant differences between treatment group in post hoc multiple comparisons (dCBTi-therapist vs dSH, *P*=.01; dCBTi-chatbot vs dSH, *P*=.04).

^j^PHQ-9: Patient Health Questionnaire-9.

^k^Significant differences between treatment group in post hoc multiple comparisons (dCBTi-assistant vs dSH, *P*=.03).

^l^GAD-7: Generalized Anxiety Disorder-7.

^m^Significant differences between treatment group in post hoc multiple comparisons (dCBTi-assistant vs dSH, *P*=.05).

^n^FAS: Fatigue Assessment Scale.

^o^ESS: Epworth Sleepiness Scale.

^p^SWLS: Satisfaction With Life Scale.

^q^DBAS-16: Dysfunctional Beliefs and Attitudes About Sleep-16.

^r^Significant differences between treatment group in post hoc multiple comparisons (dCBTi-therapist vs dSH, *P*<.001; dCBTi-assistant vs dSH, *P*=.008; dCBTi-chatbot vs dSH, *P*=.02).

^s^SRBQ: Sleep-Related Behaviors Questionnaire.

^t^Significant differences between treatment group in post hoc multiple comparisons (dCBTi-assistant vs dSH, *P*=.02).

### Treatment Efficacy

#### Hypothesis 1: dCBTi, Regardless of the Presence of Coaching, Would Be Efficacious for Improving Insomnia Symptoms Compared to dSH

**Figure 2:**
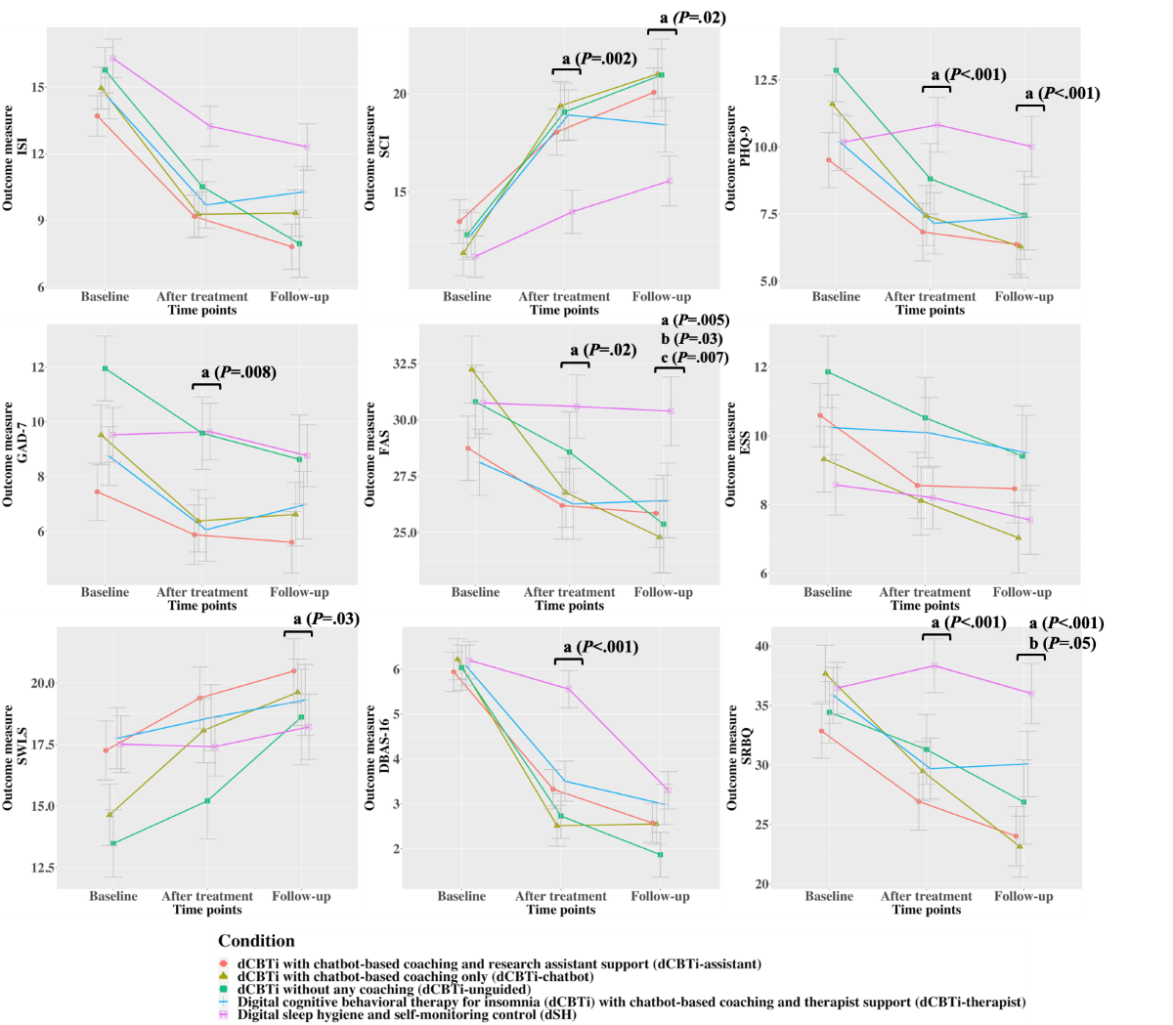
Changes in outcomes from baseline to follow-up. Error bars indicate the SEs, “a” indicates significant group-by-time effects of digital cognitive behavioral therapy for insomnia (dCBTi) versus digital sleep hygiene and self-monitoring control (dSH), “b” indicates significant group-by-time effects of dCBTi with human (therapist or research assistant) support versus dCBTi with chatbot-based coaching only (dCBTi-chatbot), and “c” indicates significant group-by-time effects of dCBTi with chatbot-based coaching and therapist support (dCBTi-therapist) versus dCBTi with chatbot-based coaching and research assistant support (dCBTi-assistant). DBAS-16: Dysfunctional Beliefs and Attitudes About Sleep-16; ESS: Epworth Sleepiness Scale; FAS: Fatigue Assessment Scale; GAD-7: Generalized Anxiety Disorder-7; ISI: Insomnia Symptom Index; PHQ-9: Patient Health Questionnaire-9; SCI: Sleep Condition Indicator; SRBQ: Sleep-Related Behaviors Questionnaire; SWLS: Satisfaction With Life Scale.

As shown in Table 4, the condition-by-time interaction effects on the SCI, PHQ-9, GAD-7, FAS, DBAS-16, and SRBQ scores were significant, indicating that participants who received dCBTi had greater improvements in insomnia symptoms measured by the SCI and greater reductions in fatigue, depressive symptoms, anxiety symptoms, dysfunctional thoughts about sleep, and safety behaviors related to sleep than participants who received dSH (Figure 2). At follow-up, significant condition-by-time interaction effects were observed on the SCI, FAS, PHQ-9, SWLS, and SRBQ scores (Table 4), indicating greater improvements in these outcomes experienced by dCBTi recipients compared to dSH recipients (Figure 2). In addition, as shown in Table 5, the remission rate based on the ISI scores was 58% (45/77) in the dCBTi conditions, which was significantly greater than that in the dSH condition (6/27, 22%). The remission rate based on the SCI score was 36% (28/77) in the dCBTi conditions, which was significantly greater than that in the dSH condition (2/27, 7%). No significant differences were observed for the rates of achieving clinically meaningful differences in the PHQ-9, GAD-7, FAS, and ESS scores.

**Table 4:** Linear mixed models results.

Outcome measures and assessment time points		Interaction effects for dCBTi conditions^a^ vs dSH^b^				Interaction effects for guided dCBTi conditions^c^ vs dCBTi-unguided				Interaction effects for dCBTi with human support^d^ vs dCBTi-chatbot				Interaction effects for dCBTi-therapist vs dCBTi-assistant		
		Estimate	*P* value	Cohen *d* (95% CI)	Estimate		*P* value	Cohen *d* (95% CI)	Estimate		*P* value	Cohen *d* (95% CI)	Estimate		*P* value	Cohen *d* (95% CI)
**ISI^e^**																
	After treatment	–2.01	.06	–0.28 (–0.42 to –0.14)	0.24		.86	0.02 (–0.08 to 0.13)	–0.30		.81	–0.03 (–0.15 to 0.08)	–1.16		.41	–0.12 (–0.22 to –0.02)
	Follow-up	–1.89	.12	–0.22 (–0.34 to –0.11)	2.57		.12	0.22 (0.13 to 0.30)	–1.54		.26	–0.16 (*–*0.27 to *–*0.06)	0.26		.86	0.03 (*–*0.07 to 0.12)
**SCI^f^**																
	After treatment	*3.83^g^*	*.003*	*0.45 (0.33 to 0.56)*	–0.20		.90	–0.02 (*–*0.11 to 0.07)	–0.08		.96	–0.01 (*–*0.10 to 0.09)	2.94		.08	0.26 (0.17 to 0.34)
	Follow-up	*3.51*	*.02*	0.35 (0.24 to 0.45)	–1.03		.60	–0.07 (*–*0.140 to *–*0.002)	2.25		.17	0.20 (0.11 to 0.29)	2.54		.15	0.21 (0.13 to 0.29)
**PHQ-9^h^**																
	After treatment	–*4.12*	*<.001*	–*0.62 (–0.77 to* *–0.47)*	0.75		.55	0.09 (*–*0.03 to 0.21)	–0.40		.74	–0.05 (*–*0.18 to 0.08)	–1.47		.26	–0.17 (*–*0.29 to *–*0.06)
	Follow-up	–*4.00*	*<.001*	–*0.52 (–0.65 to* *–0.39)*	1.65		.31	0.15 (0.06 to 0.24)	–1.44		.26	–0.17 (*–*0.29 to *–*0.05)	–2.16		.11	–0.24 (*–*0.35 to *–*0.13)
**GAD-7^i^**																
	After treatment	–*2.56*	*.01*	–*0.40 (–0.56 to* *–0.25)*	–0.10		.93	–0.01 (*–*0.13 to 0.11)	0.33		.77	0.04 (*–*0.09 to 0.17)	–1.57		.21	–0.19 (*–*0.31 to *–*0.07)
	Follow-up	–1.70	.12	–0.23 (–0.37 to –0.09)	1.14		.46	0.11 (0.01 to 0.20)	–0.58		.63	–0.07 (–0.19 to 0.05)	–1.06		.42	–0.12 (–0.24 to –0.01)
**FAS^j^**																
	After treatment	–*2.87*	*.02*	–*0.35 (–0.47 to –0.23)*	–1.05		.50	–0.10 (–0.200 to –0.004)	–2.15		.14	–0.22 (–0.32 to –0.12)	–2.93		.07	–0.28 (–0.37 to –0.18)
	Follow-up	–*4.01*	*.01*	–*0.42 (–0.53 to –0.33)*	1.43		.47	0.11 (0.03 to 0.18)	–*3.47*		*.03*	–*0.33 (–0.43 to –0.24)*	–*4.56*		*.01*	–*0.41 (–0.50 to –0.32)*
**ESS^k^**																
	After treatment	–0.81	.35	–0.14 (–0.31 to 0.03)	0.20		.85	0.03 (–0.11 to 0.16)	–1.47		.16	–0.21 (–0.36 to –0.07)	0.83		.47	0.11 (–0.02 to 0.24)
	Follow-up	–0.88	.38	–0.13 (–0.28 to 0.02)	0.73		.61	0.08 (–0.03 to 0.18)	–1.48		.18	–0.20 (–0.33 to –0.06)	–0.15		.90	–0.02 (–0.15 to 0.11)
**SWLS^l^**																
	After treatment	2.14	.07	0.27 (0.14 to 0.39)	0.40		.79	0.04 (–0.06 to 0.14)	1.94		.17	0.20 (0.10 to 0.31)	1.30		.40	0.13 (0.03 to 0.22)
	Follow-up	*3.02*	*.03*	*0.33 (0.22 to 0.43)*	–1.88		.33	–0.14 (–0.22 to –0.07)	2.55		.10	0.25 (0.15 to 0.35)	1.75		.28	0.16 (0.07 to 0.25)
**DBAS-16^m^**																
	After treatment	–*2.41*	*<.001*	–*0.53 (–0.75 to –0.31)*	0.34		.62	0.06 (–0.12 to 0.25)	–0.58		.39	–0.11 (–0.30 to 0.08)	–1.09		.16	–0.18 (–0.34 to –0.02)
	Follow-up	–0.68	.24	–0.15 (–0.37 to 0.07)	0.79		.25	0.15 (–0.04 to 0.33)	–0.42		.53	–0.08 (–0.27 to 0.11)	–0.30		.70	–0.05 (–0.21 to 0.11)
**SRBQ^n^**																
	After treatment	–*7.74*	*.001*	–*0.50 (–0.57 to –0.43)*	–3.64		.21	–0.18 (–0.23 to –0.13)	–0.90		.74	–0.05 (–0.10 to 0.01)	–2.30		.45	–0.11 (–0.16 to –0.06)
	Follow-up	–*8.71*	*.001*	–*0.49 (–0.54 to –0.43)*	–2.17		.54	–0.09 (–0.13 to –0.05)	–*5.93*		*.05*	–*0.30 (–0.35 to –0.25)*	–5.70		.07	–0.27 (–0.32 to –0.22)

^a^Digital cognitive behavioral therapy for insomnia (dCBTi) with chatbot-based coaching and therapist support (dCBTi-therapist), dCBTi with chatbot-based coaching and research assistant support (dCBTi-assistant), dCBTi with chatbot-based coaching only (dCBTi-chatbot), and dCBTi without any coaching (dCBTi-unguided).

^b^dSH: digital sleep hygiene and self-monitoring control.

^c^dCBTi-therapist, dCBTi-assistant, and dCBTi-chatbot.

^d^dCBTi-therapist and dCBTi-assistant.

^e^ISI: Insomnia Severity Index.

^f^SCI: Sleep Condition Indicator.

^g^Italicization refers to significant results. See the respective columns of *P* value.

^h^PHQ-9: Patient Health Questionnaire-9.

^i^GAD-7: Generalized Anxiety Disorder-7.

^j^FAS: Fatigue Assessment Scale.

^k^ESS: Epworth Sleepiness Scale.

^l^SWLS: Satisfaction With Life Scale.

^m^DBAS-16: Dysfunctional Beliefs and Attitudes About Sleep-16.

^n^SRBQ: Sleep-Related Behaviors Questionnaire.

**Table 5:** Comparison of the rates of remission or clinically meaningful changes across conditions after treatment.

Outcome	Comparison between dCBTi conditions^a^ and dSH^b^				Comparison between guided dCBTi conditions^c^ and dCBTi-unguided				Comparison between dCBTi with human support^d^ and dCBTi-chatbot					Comparison between dCBTi-therapist and dCBTi-assistant			
	dCBTi conditions (n=77), n (%)	dSH (n=27), n (%)	Chi-square (*df*)	*P* value	Guided dCBTi conditions (n=63), n (%)	dCBTi-unguided (n=14), n (%)	Chi-square (*df*)	*P* value	dCBTi with human support (n=44), n (%)	dCBTi-chatbot (n=19), n (%)	Chi-square (*df*)	*P* value	dCBTi-therapist (n=21), n (%)		dCBTi-assistant (n=23), n (%)	Chi-square (*df*)	*P* value
ISI^e^	*45 (58.44)* ^f^	*6 (22.22)*	*9.1* (1)	*.003*	37 (58.73)	8 (57.14)	<0.1 (1)	.99	26 (59.09)	11 (57.89)	<0.1 (1)	.99	13 (61.90)		13 (56.52)	<0.1 (1)	.96
SCI^g^	*28 (36.36)*	*2 (7.41)*	*6.8* (1)	*.01*	22 (42.86)	6 (34.92)	0.1 (1)	.80	15 (34.09)	7 (36.84)	<0.01 (1)	.99	8 (38.10)		7 (30.43)	0.1 (1)	.83
PHQ-9^h^	28 (36.84)	4 (16)	2.9 (1)	.09	21 (33.87)	7 (50)	0.7 (1)	.41	17 (38.64)	4 (22.22)	0.9 (1)	.35	10 (47.62)		7 (30.43)	0.7 (1)	.39
GAD-7^i^	25 (32.89)	5 (20)	0.9 (1)	.33	20 (32.36)	5 (35.71)	<0.1 (1)	.99	13 (29.55)	7 (38.89)	0.2 (1)	.68	*10 (47.62)*		*3 (13.04)*	*4.8* (1)	*.03*
FAS^j^	35 (46.05)	8 (32)	1.0 (1)	.32	30 (48.39)	5 (35.71)	0.3 (1)	.57	22 (50)	8 (44.44)	<0.1 (1)	.91	13 (61.90)		9 (39.13)	1.5 (1)	.23
ESS^k^	33 (43.42)	6 (24)	2.2 (1)	.14	28 (45.16)	5 (35.71)	0.1 (1)	.73	22 (50)	6 (33.33)	0.8 (1)	.36	10 (47.62)		12 (52.17)	<0.1 (1)	.99

^a^Digital cognitive behavioral therapy for insomnia (dCBTi) with chatbot-based coaching and therapist support (dCBTi-therapist), dCBTi with chatbot-based coaching and research assistant support (dCBTi-assistant), dCBTi with chatbot-based coaching only (dCBTi-chatbot), and dCBTi without any coaching (dCBTi-unguided).

^b^dSH: digital sleep hygiene and self-monitoring control.

^c^dCBTi-therapist, dCBTi-assistant, and dCBTi-chatbot.

^d^dCBTi-therapist and dCBTi-assistant.

^e^ISI: Insomnia Severity Index (criterion of remission: ISI score <10).

^f^Italicization refers to significant results. See the respective columns of *P* value.

^g^SCI: Sleep Condition Indicator (criterion of remission: SCI score >21).

^h^PHQ-9: Patient Health Questionnaire-9 (criterion of reaching clinically meaningful difference: 5-point change).

^i^GAD-7: Generalized Anxiety Disorder-7 (criterion of reaching clinically meaningful difference: 4-point change).

^j^FAS: Fatigue Assessment Scale (criterion of reaching clinically meaningful difference: 4-point change).

^k^ESS: Epworth Sleepiness Scale (criterion of reaching clinically meaningful difference: 2-point change).

#### Hypothesis 2: dCBTi With Coaching Would Promote Greater Improvements in Insomnia Symptoms and Greater Treatment Adherence Than dCBTi-Unguided

No significant interaction effects were found on all outcomes after treatment and at follow-up when comparing guided dCBTi and dCBTi-unguided (Table 4), suggesting that adding chatbot-based coaching and human support did not improve treatment efficacy. Similarly, the rates of remission of insomnia and the rates of achieving clinically meaningful changes in the secondary outcomes did not differ significantly between guided dCBTi and dCBTi-unguided (Table 5).

#### Hypothesis 3: dCBTi With Human Coaching Would Promote Greater Improvements in Insomnia Symptoms and Treatment Adherence Than dCBTi-Chatbot

Significant condition-by-time interaction effects were observed on the FAS and SRBQ scores at follow-up (Table 4), indicating that participants who received dCBTi-therapist or dCBTi-assistant experienced greater reductions in fatigue and sleep-related safety behaviors than those who received dCBTi-chatbot (Figure 2). The rates of remission of insomnia and the rates of achieving clinically meaningful changes in the secondary outcomes did not differ significantly between dCBTi with human support and dCBTi-chatbot (Table 5).

#### Hypothesis 4: dCBTi-Therapist Would Promote Greater Improvements in Insomnia Symptoms and Greater Treatment Adherence Than dCBTi-Assistant

A significant condition-by-time interaction effect was observed on the FAS scores at follow-up (Table 4), indicating that participants who received dCBTi-therapist experienced greater reductions in fatigue than those who received dCBTi-assistant (Figure 2). In addition, the rate of achieving clinically meaningful changes in the GAD-7 scores was significantly greater in dCBTi-therapist than in dCBTi-assistant (Table 5).

### Treatment Adherence

Table 6 presents the results of treatment adherence across the conditions. As expected, participants in dCBTi-therapist and dCBTi-assistant completed significantly more video sessions than participants in dCBTi-unguided. They also completed more weeks of sleep diaries than participants in dSH. Significant differences in treatment adherence were observed especially in later sessions, with more participants in dCBTi-therapist completing sleep diaries during sessions 4 to 6 compared to those in dCBTi-unguided and dSH.

**Table 6:** Video completion and sleep diary completion across conditions.

Variables			dCBTi-therapist^a^ (n=25)		dCBTi-assistant^b^ (n=27)		dCBTi-chatbot^c^ (n=26)		dCBTi-unguided^d^ (n=21)		dSH^e^ (n=30)		*F* test (*df*)		*P* value
**Video sessions completed**															
	Total, mean (SD)	4.24 (1.83)^f^		3.37 (2.50)^f^		2.69 (2.24)		1.48 (2.02)^f^		—^g^		6.58 (3,95)		<.001	
	Session 1, n (%)	20 (80)^f^		18 (67)		15 (56)		10 (48)^f^		—		—		.03	
	Session 2, n (%)	19 (76)		18 (67)		14 (54)		9 (43)		—		—		.09	
	Session 3, n (%)	21 (84)^f^		17 (63)		12 (46)^f^		6 (29)^f^		—		—		<.001	
	Session 4, n (%)	18 (72)		14 (52)		12 (46)		5 (24)		—		—		.10	
	Session 5, n (%)	16 (64)^f^		14 (52)		10 (38)		5 (24)^f^		—		—		.01	
	Session 6, n (%)	16 (64)^f^		13 (48)^f^		8 (32)^f^		3 (14)^f^		—		—		<.001	
**Weeks of diaries completed**															
	Total, mean (SD)	5.32 (1.55)^h^		4.74 (1.87)		3.96 (2.57)		3.81 (2.18)		3.60 (2.33)^h^		2.95 (4,124)		.02	
	Week 1, n (%)	24 (96)		24 (89)		21 (81)		19 (90)		27 (90)		—		.57	
	Week 2, n (%)	21 (84)		25 (93)		19 (73)		17 (81)		22 (73)		—		.31	
	Week 3, n (%)	22 (88)		21 (78)		17 (65)		14 (67)		18 (60)		—		.15	
	Week 4, n (%)	22 (88)^h^		21 (78)		16 (62)		12 (571)		15 (50)^h^		—		.02	
	Week 5, n (%)	22 (88)^h^		18 (67)		16 (62)		10 (48)^h^		14 (47)^h^		—		.01	
	Week 6, n (%)	22 (88)^h^		19 (70)		14 (54)		8 (38)^h^		12 (39)^h^		—		.001	

^a^dCBTi-therapist: digital cognitive behavioral therapy for insomnia with chatbot-based coaching and therapist support.

^b^dCBTi-assistant: digital cognitive behavioral therapy for insomnia with chatbot-based coaching and research assistant support.

^c^dCBTi-chatbot: digital cognitive behavioral therapy for insomnia with chatbot-based coaching only.

^d^dCBTi-unguided: digital cognitive behavioral therapy for insomnia without any coaching.

^e^dSH: digital sleep hygiene and self-monitoring control.

^f^Significant differences between treatment groups in the same row in post hoc multiple comparisons with adjustments for multiple tests (dCBTi-therapist vs dCBTi-unguided, *P*<.001; dCBTi-assistant vs dCBTi-unguided, *P*=.02).

^g^Not applicable.

^h^Significant differences between treatment groups (dCBTi-therapist vs dSH, *P*=.03).

## Discussion

### Principal Findings

This work presents the first randomized controlled comparative trial that evaluates the effects of chatbot-based coaching and human support on the treatment efficacy of, and adherence to, dCBTi. We found that participants who received dCBTi had greater improvements in insomnia symptoms (measured using the SCI), mood disturbances, fatigue, and life satisfaction as well as greater reductions in dysfunctional beliefs and safety behaviors related to insomnia than those who received dSH, with medium effect sizes comparable to those in previous studies of dCBTi [62-65]. Most of the improvements in the dCBTi conditions were sustained at 4-week follow-up. Surprisingly, adding chatbot-based coaching and human support did not significantly improve treatment effects on insomnia. Nonetheless, adding human support, especially therapist support, promoted greater improvements in fatigue as well as greater reduction in safety behaviors related to sleep. Adding chatbot-based coaching and human support also improved some indicators of treatment adherence.

### Does Fully Automated dCBTi-Unguided Work?

Supporting hypothesis 1, dCBTi delivered by a fully automated mobile app is efficacious for improving insomnia, mood disturbances, fatigue, and quality of life in adults with insomnia, with effect sizes comparable to those of other tested versions of dCBTi [62-65]. Recipients of dCBTi, regardless of having coaching, achieved an average increase of 12% (SD 13.43%) in sleep efficiency after treatment, from 75.4% to 87.4%; noting that ≥85% sleep efficiency is considered remission of insomnia [45]. The remission rate reached 58% (45/77) in dCBTi conditions compared to 22% (6/27) in dSH. This study was one of the few randomized controlled trials of dCBTi conducted in non-Western populations, and this was the only dCBTi mobile app implemented in Cantonese with published efficacy. This study also extended previous findings by showing that dCBTi was also efficacious for reducing dysfunctional beliefs about sleep and maladaptive behaviors related to sleep—the mechanisms theorized to bring about the treatment effects in CBTi. This finding provided even stronger support for dCBTi by showing that it worked in a way that was consistent with the theory.

Unexpectedly, a greater reduction in insomnia symptoms in the dCBTi group was only reflected by the SCI scores but not the ISI scores. The SCI differs from the ISI in that its ratings on sleep difficulties are based on the recommended quantitative criteria from the *DSM-5* as opposed to qualitative impressions of insomnia symptom severity. The inconsistent results reflected by the scores on the two scales might suggest that quantitative anchors are more sensitive in detecting changes in insomnia symptoms. Nevertheless, the absence of a treatment effect of dCBTi on the ISI scores in this study differed from the findings of previous studies [62,65,66]. These previous studies used more stringent participant inclusion criteria; for instance, in addition to scoring ≥10 on the ISI, participants had to meet the duration (≥3 mo) and frequency (≥3 d/wk) diagnostic criteria of insomnia disorder. Participants in these studies might have had more chronic and severe insomnia to begin with and hence experienced greater improvements. Indeed, the ISI scores were 17 [65] and 19 [62] in prior studies and 15 in our sample. Our sample might have also included individuals with acute insomnia. As acute and subclinical insomnia could predict chronic insomnia and depressive episodes [67,68], the evidence for the efficacy of dCBTi for this group of participants with a potentially wider range of symptom duration and severity adds confidence for the impact of dCBTi at the population level where people with differing symptom duration and severity could benefit from dCBTi.

### Does Adding Chatbot-Based Coaching and Human Support Improve dCBTi?

Partially consistent with hypothesis 2, adding coaching support improved treatment adherence to dCBTi but not efficacy. Our findings suggested that both human-assisted guidance and chatbot-based coaching were useful strategies to enhance engagement in the middle and late stages of dCBTi. The treatment adherence rates in the guided dCBTi conditions were double those of dCBTi-unguided. Among all types of guided dCBTi, dCBTi-therapist had the highest adherence rate, followed by dCBTi-assistant and dCBTi-chatbot. However, increased adherence rates were not associated with greater efficacy. Consistent with previous meta-analyses [24,69,70] showing that dCBTi was not inferior to face-to-face CBTi for alleviating insomnia, this study did not find any meaningful differences (a change of >4 points in the ISI total score) between guided CBTi conditions and dCBTi-unguided. This study was the first direct comparison of guided CBTi and dCBTi-unguided with different types of guidance and provided primary evidence indicating that adding either therapist or research assistant support does not promote meaningfully greater treatment efficacy.

It is possible that the high degrees of personalization offered by dCBTi might have minimized the benefit of coaching support on treatment efficacy; for instance, in the dCBTi-unguided condition, participants still received a tailored sleep schedule suggestion based on their diary-reported sleep data in the previous week. They were also prompted to set up individualized weekly goals and action plans. With mobile technology, even dCBTi-unguided could deliver tailored treatment recommendations, which is one of the promising benefits of dCBTi. Furthermore, our sample might have included participants with acute insomnia, and the insomnia symptoms experienced by these individuals might not necessitate coaching support. In addition, our sample is overrepresented by highly educated young adults (104/129, 80.6%) with few psychiatric comorbidities. Coaching support may not be most needed for this population; adding therapist support may be beneficial specifically for patients with psychiatric comorbidities [71]. Nevertheless, the differences in adherence between guided dCBTi and dCBTi-unguided reflected the utility of coaching support for enhancing engagement and potentially reducing early dropouts and motivational barriers.

Similarly, partially consistent with hypothesis 3, adding human support did not promote greater improvements in insomnia and most outcomes compared to dCBTi-chatbot. However, greater improvements in fatigue and greater reductions in safety behaviors related to sleep were observed in dCBTi with human support compared to dCBTi-chatbot. These incremental benefits might promote greater or more sustained improvements in sleep and well-being in the long term because reduced fatigue and safety behaviors related to sleep could potentially enhance the maintenance of positive changes resulting from dCBTi, such as maintaining adequate daytime activities and inhibiting anxiety and frustration about sleep. Indeed, as shown in a previous study, adding human support to a self-help CBTi did not lead to greater improvements in insomnia symptoms after treatment and at 4-week follow-up; however, the incremental improvements appeared later at the 3-month follow-up [29].

### Is Support From a Therapist Better Than That From a Research Assistant?

Inconsistent with hypothesis 4, support from a therapist did not promote superior treatment efficacy for most outcomes or superior treatment adherence compared to support from a research assistant. While this study was the first to directly compare human therapist support and research assistant support in dCBTi, our results converged with a prior study on digital intervention for depression to suggest that treatment efficacy was comparable between therapist-guided and nontherapist-guided digital interventions [72]. However, it should be noted that the dCBTi-assistant telephone call completion rate was much lower than the dCBTi-therapist telephone call rate, suggesting that therapist support was much more welcomed by the participants in comparison. The lack of differences between these two conditions could be explained by the lack of statistical power for detecting smaller effects. There were no prior data on the expected effect size for the difference between dCBTi-therapist and dCBTi-assistant. This study could have missed smaller effects between these two conditions. Indeed, as shown in Table 4, differences between dCBTi-therapist and dCBTi-assistant amounting to small effect sizes were observed in the SCI, PHQ-9, GAD-7, FAS, and SRBQ scores. Future studies with larger sample sizes statistically powered to detect small effects are needed to further elucidate whether therapist support promotes incremental treatment efficacy.

### Limitations

Our findings need to be interpreted in light of the following limitations. First, our sample consisted of mostly highly educated young adults (104/129, 80.6%); therefore, the findings may not be generalizable to other populations. While dCBTi was found to be effective across demographic groups [67], all studies were conducted in samples of working-age adults [68]. It remains unclear whether older adults also respond as well to dCBTi. Second, although this study was adequately powered for detecting meaningful differences in the ISI scores, it could not detect smaller effects that might have existed in the comparison between dCBTi conditions with different coaching types. Nonetheless, we argue that such differences would have limited practical and clinical implications. Third, the follow-up assessment was conducted 4 weeks after treatment, thereby limiting any conclusions that could be drawn about the long-term efficacy of the dCBTi intervention. In particular, although we did not find significant meaningful differences in the primary outcomes between dCBTi conditions with different types of coaching at short-term follow-up, dCBTi with coaching, especially dCBTi-therapist, performed better than dCBTi without coaching on the mechanism of action, that is, sleep-related safety behaviors. This greater improvement in the mechanism of action may promote incremental benefits on the primary outcome that appear at a longer follow-up. Future studies with longer follow-up are necessary to fully evaluate the potential benefits of adding coaching to dCBTi. Finally, we did not collect data on participants’ use of strategies learned in dCBTi. Although adding therapist support improved video session and sleep diary completions, it remains unclear whether the addition of such support increased participants’ use of the learned strategies in their daily lives. More detailed assessments of adherence would provide greater insights into the relationship between treatment adherence and efficacy or the lack thereof.

### Conclusions

Our findings supported the efficacy of a fully automated dCBTi intervention, Sleep Sensei, compared to an active control for treating insomnia, reducing mood disturbances and fatigue, and improving quality of life. Adding chatbot-based coaching and human support did not significantly improve the efficacy of Sleep Sensei for treating insomnia, but doing so may improve long-term efficacy, given their effects on increasing treatment adherence and additional benefits on reducing fatigue and behaviors that could perpetuate insomnia. In sum, Sleep Sensei can be used as a stand-alone intervention for treating insomnia and is the only Cantonese mobile app for CBTi published with demonstrated efficacy.

## Data Availability

The data sets generated and analyzed during this study are available from the corresponding author on reasonable request.

## Appendix

Multimedia Appendix 1 Screenshots of the Sleep Sensei interface.

Multimedia Appendix 2 Consort Ehealth checklist.

## Abbreviations

CBTi: cognitive behavioral therapy for insomnia
CONSORT: Consolidated Standards of Reporting Trials
DBAS-16: Dysfunctional Beliefs and Attitudes About Sleep-16
dCBTi: digital cognitive behavioral therapy for insomnia
dCBTi-assistant: digital cognitive behavioral therapy for insomnia with chatbot-based coaching and research assistant support
dCBTi-chatbot: digital cognitive behavioral therapy for insomnia with chatbot-based coaching only
dCBTi-therapist: digital cognitive behavioral therapy for insomnia with chatbot-based coaching and therapist support
dCBTi-unguided: digital cognitive behavioral therapy for insomnia without any coaching
dSH: digital sleep hygiene and self-monitoring control
DSM-5: Diagnostic and Statistical Manual of Mental Disorders, Fifth Edition
ESS: Epworth Sleepiness Scale
FAS: Fatigue Assessment Scale
GAD-7: Generalized Anxiety Disorder-7
ISI: Insomnia Severity Index
PHQ-9: Patient Health Questionnaire-9
SCI: Sleep Condition Indicator
SRBQ: Sleep-Related Behaviors Questionnaire
SWLS: Satisfaction With Life Scale
TIB: time in bed
TST: total sleep time
