# Supplementary material for: Assessing the Short-Term Efficacy of Digital Cognitive Behavioral Therapy for Insomnia With Different Types of Coaching: Randomized Controlled Comparative Trial
Source: JMIR Ment Health. 2024 Aug 7;11:e51716. doi: 10.2196/51716 (PMC11339566; doi:10.2196/51716)

VIDEO LESSONS

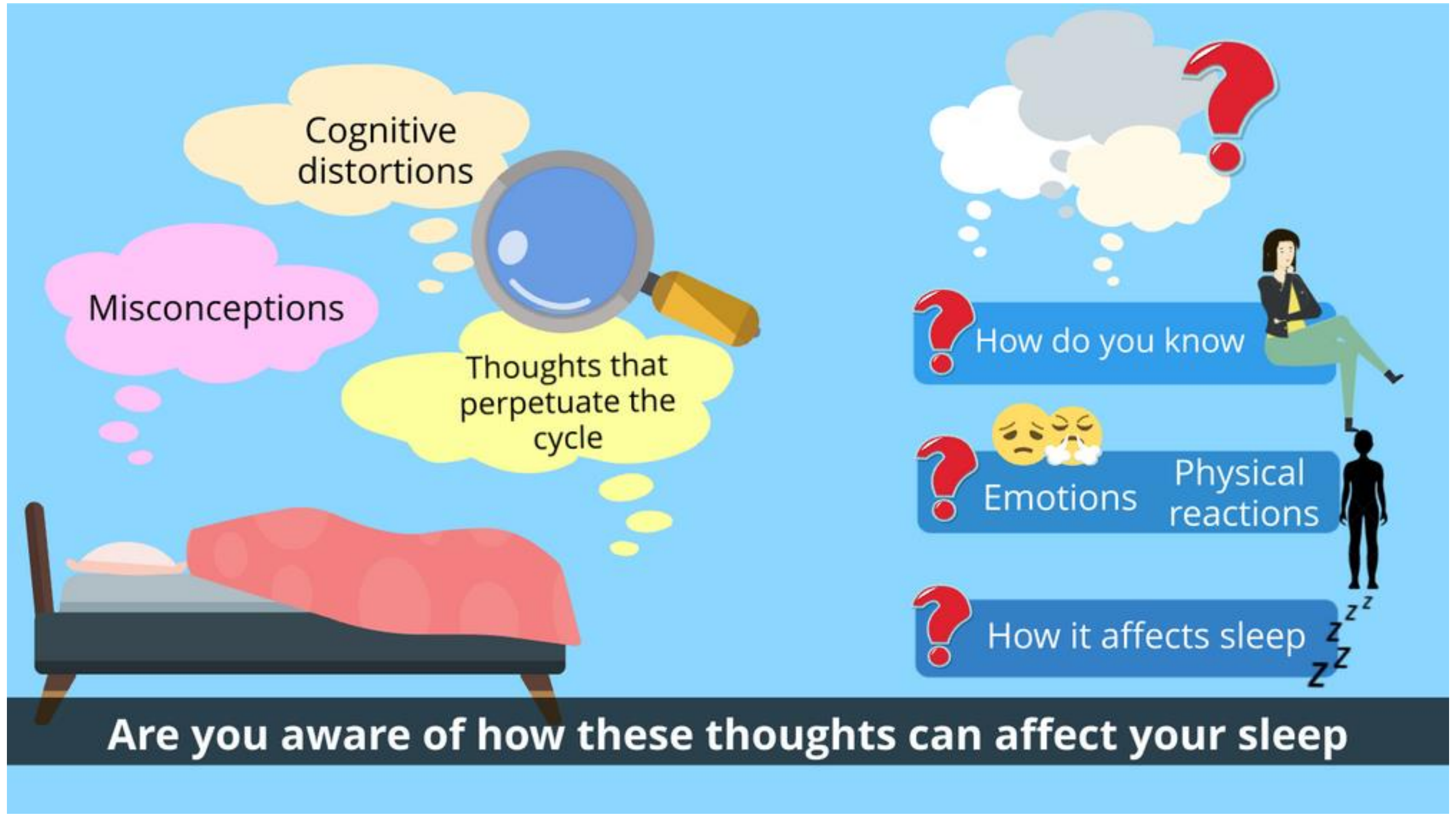

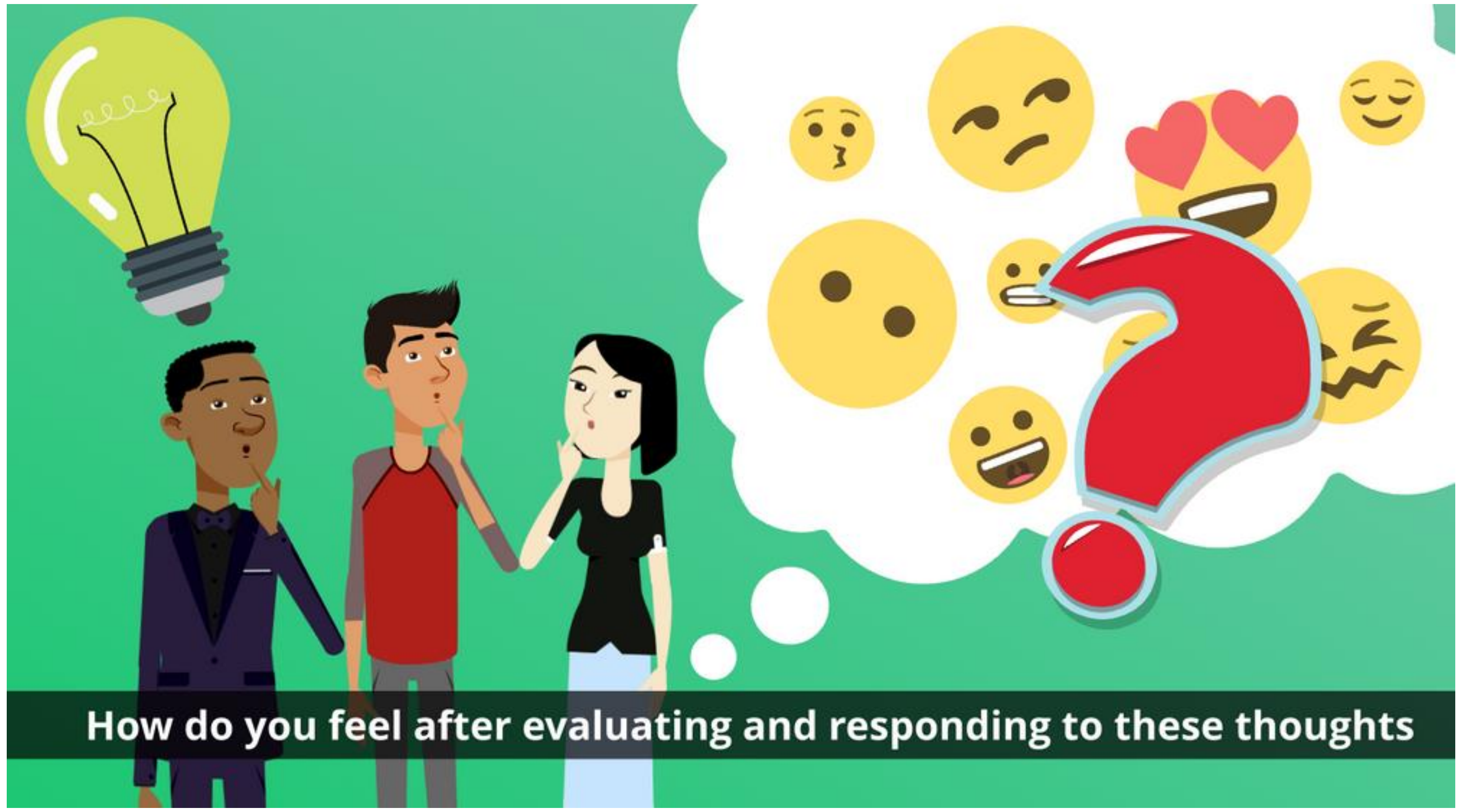

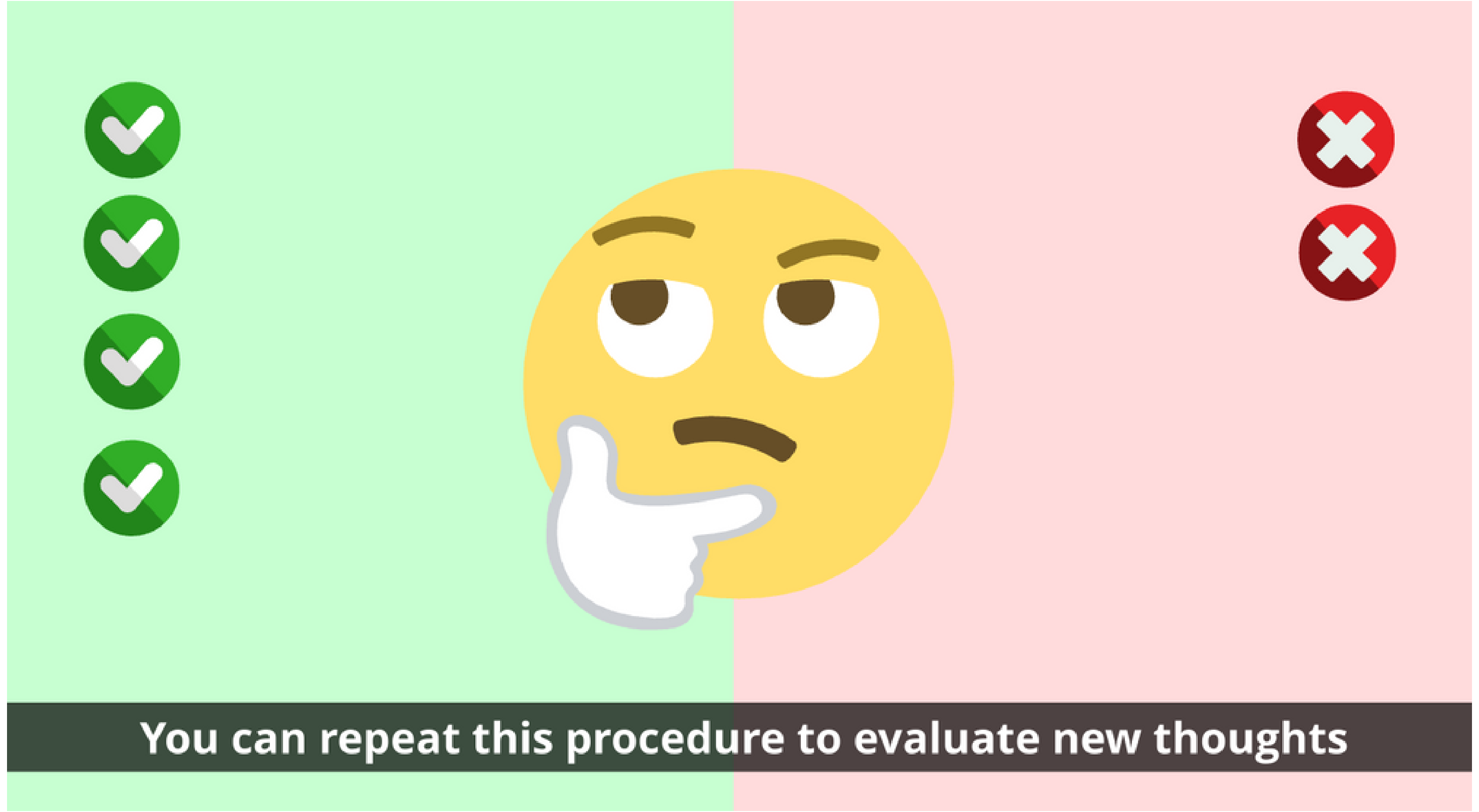

COACH DIALOGUE

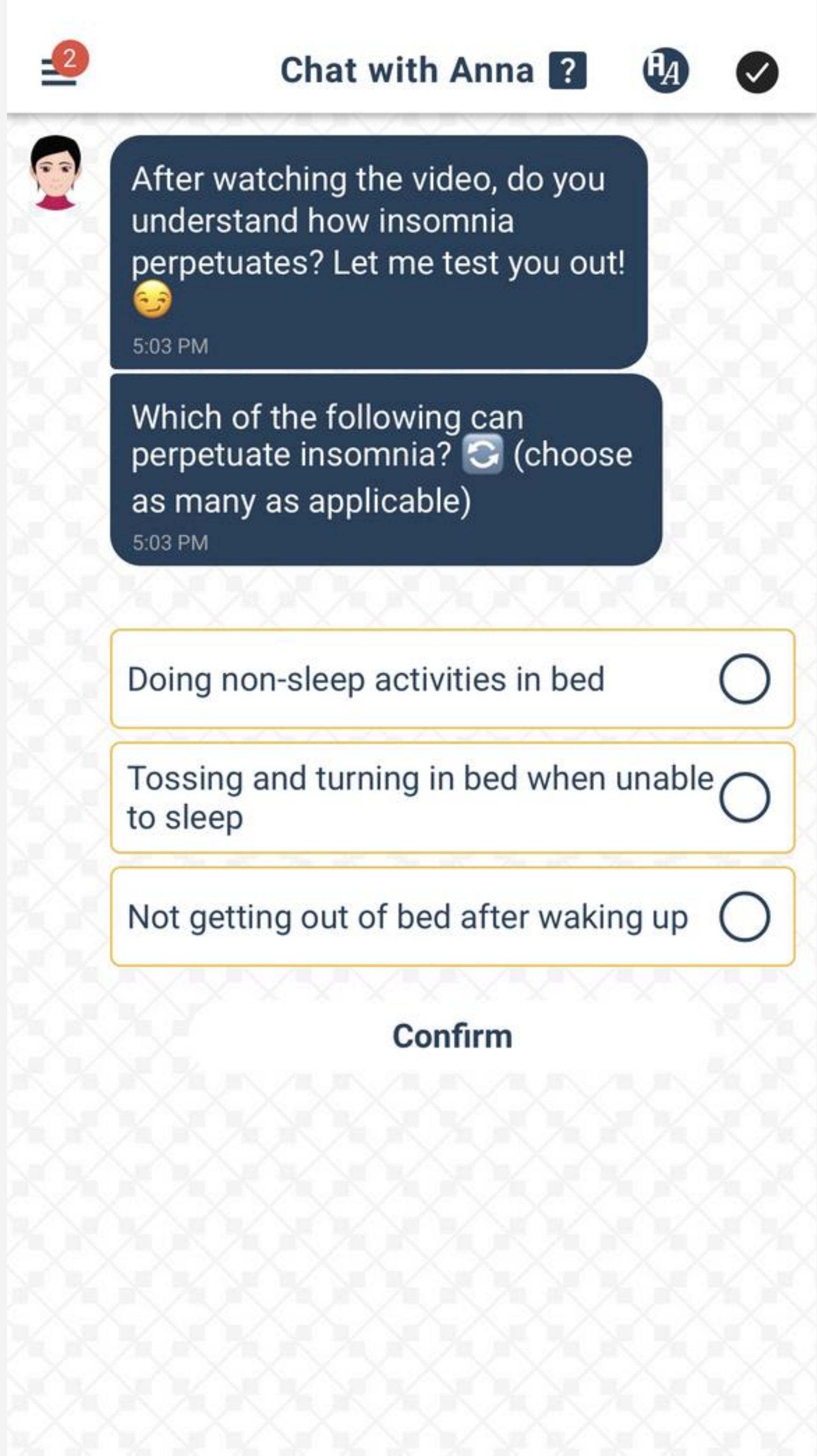

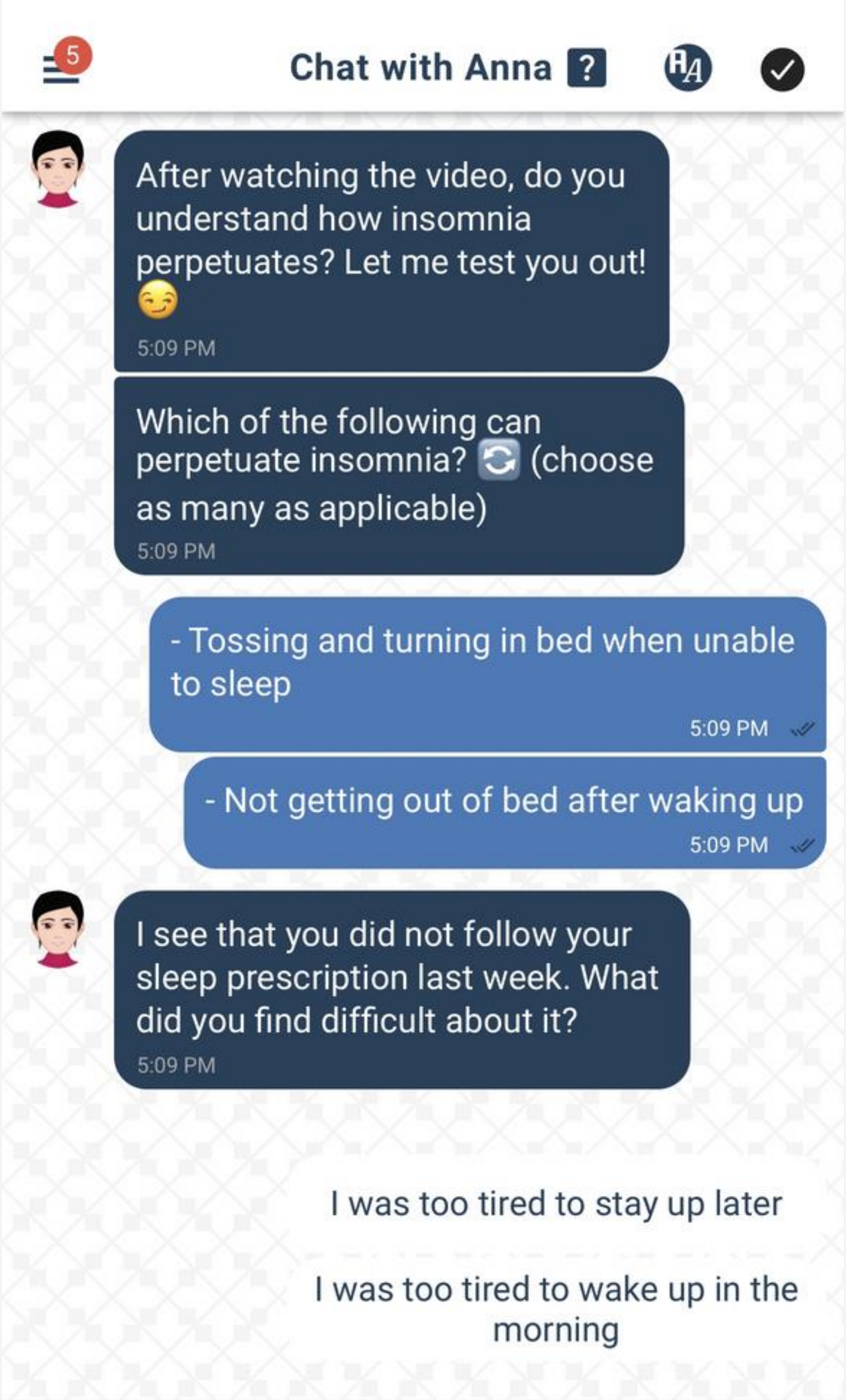

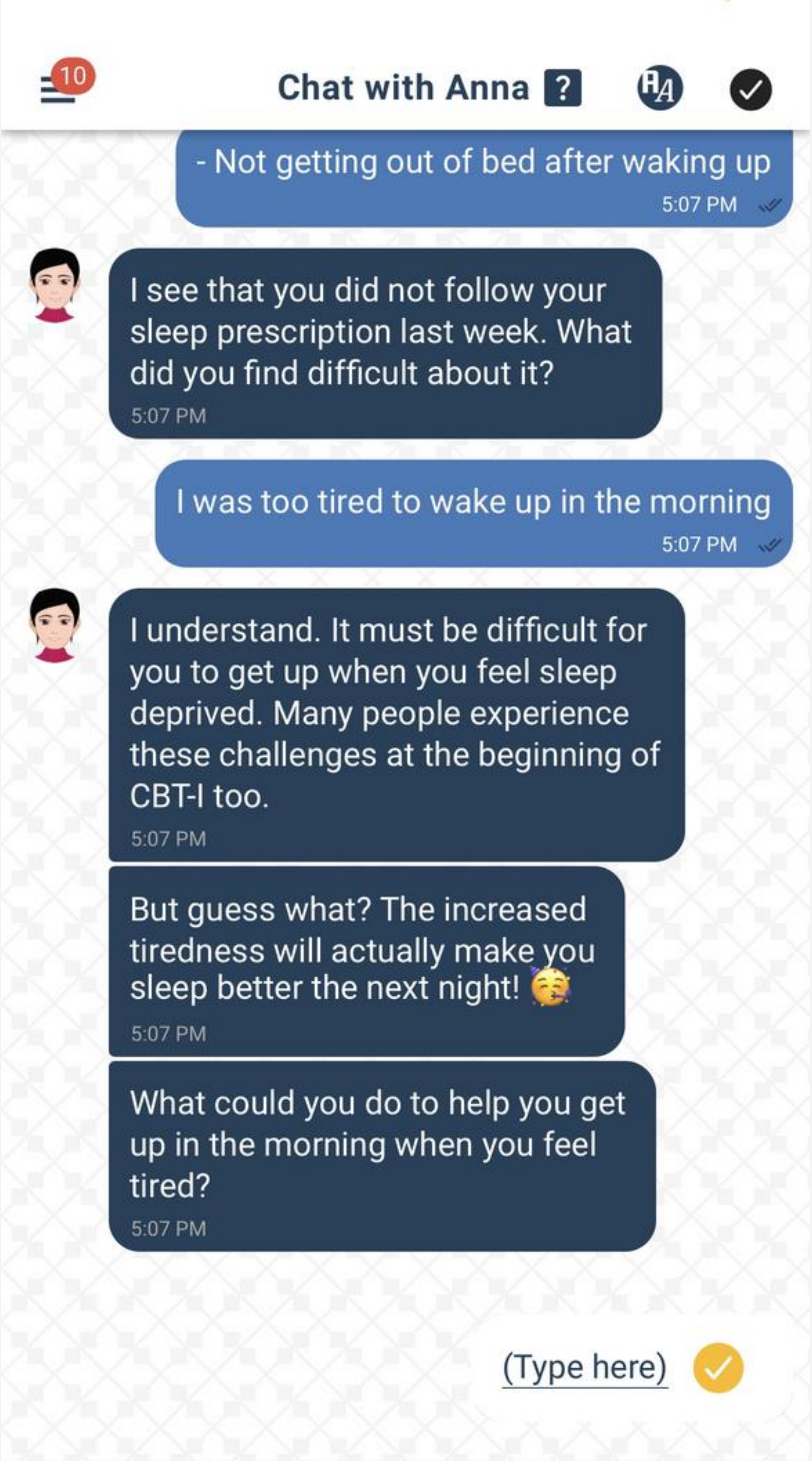

DAILY CHECK-IN

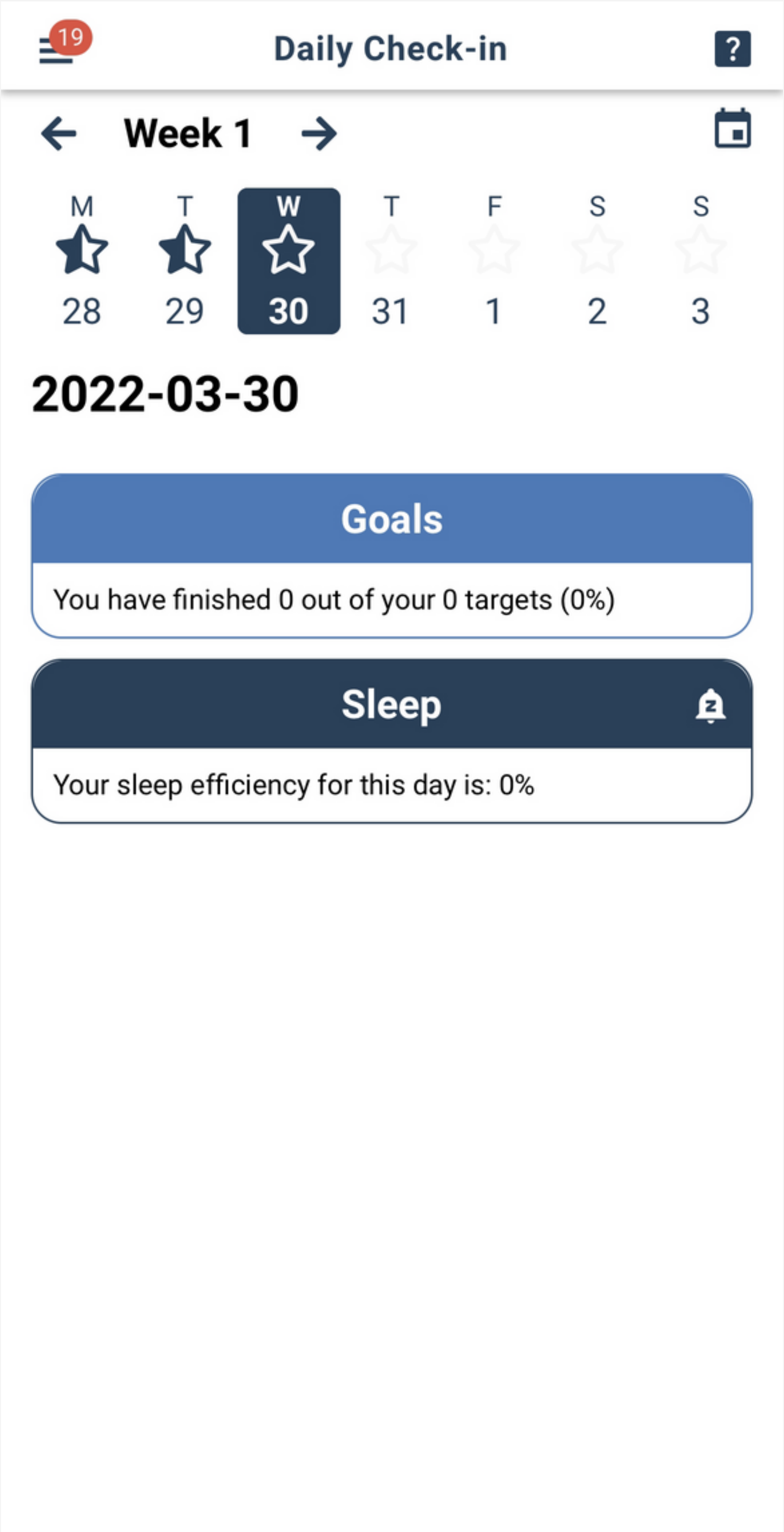

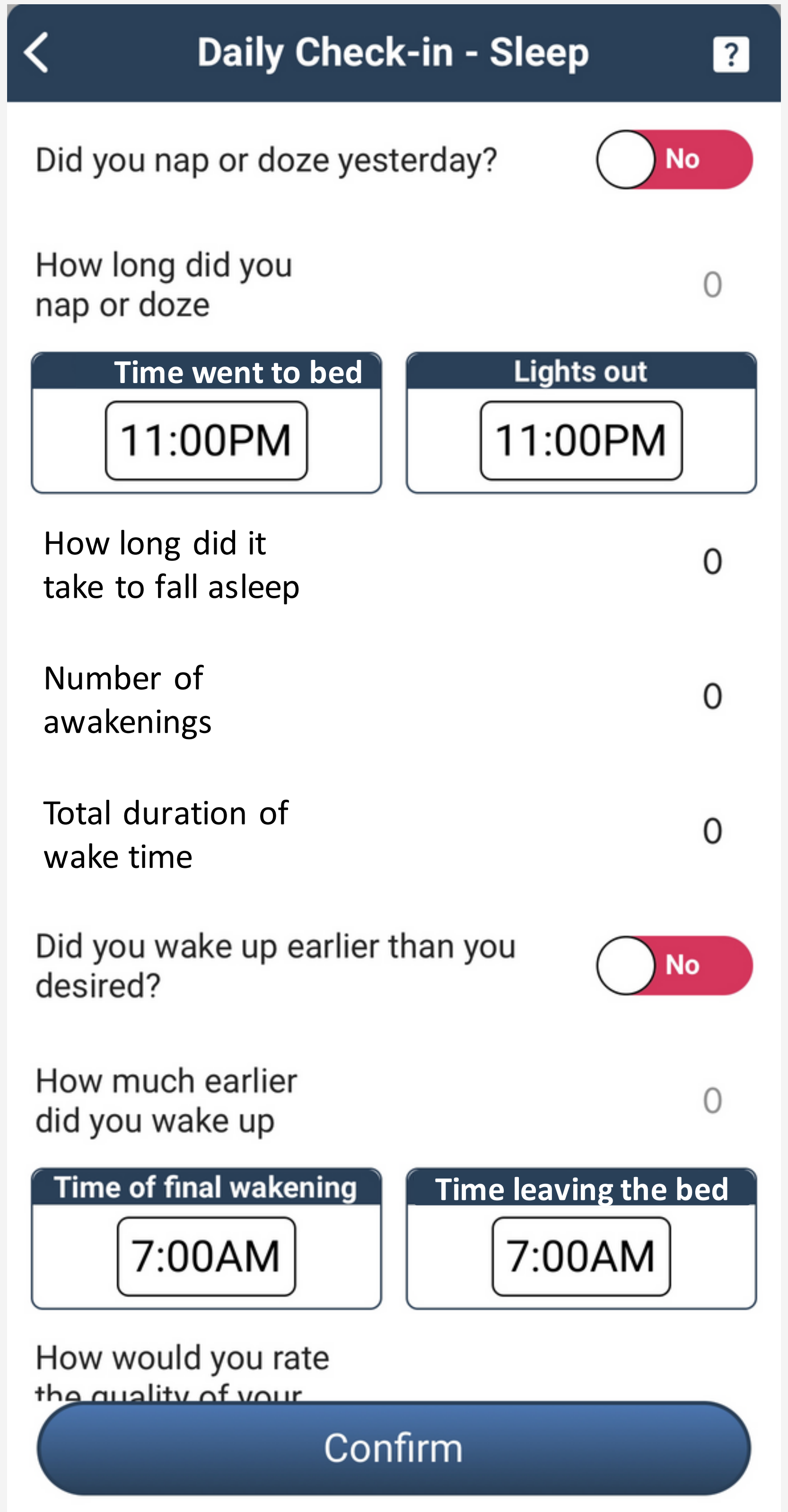

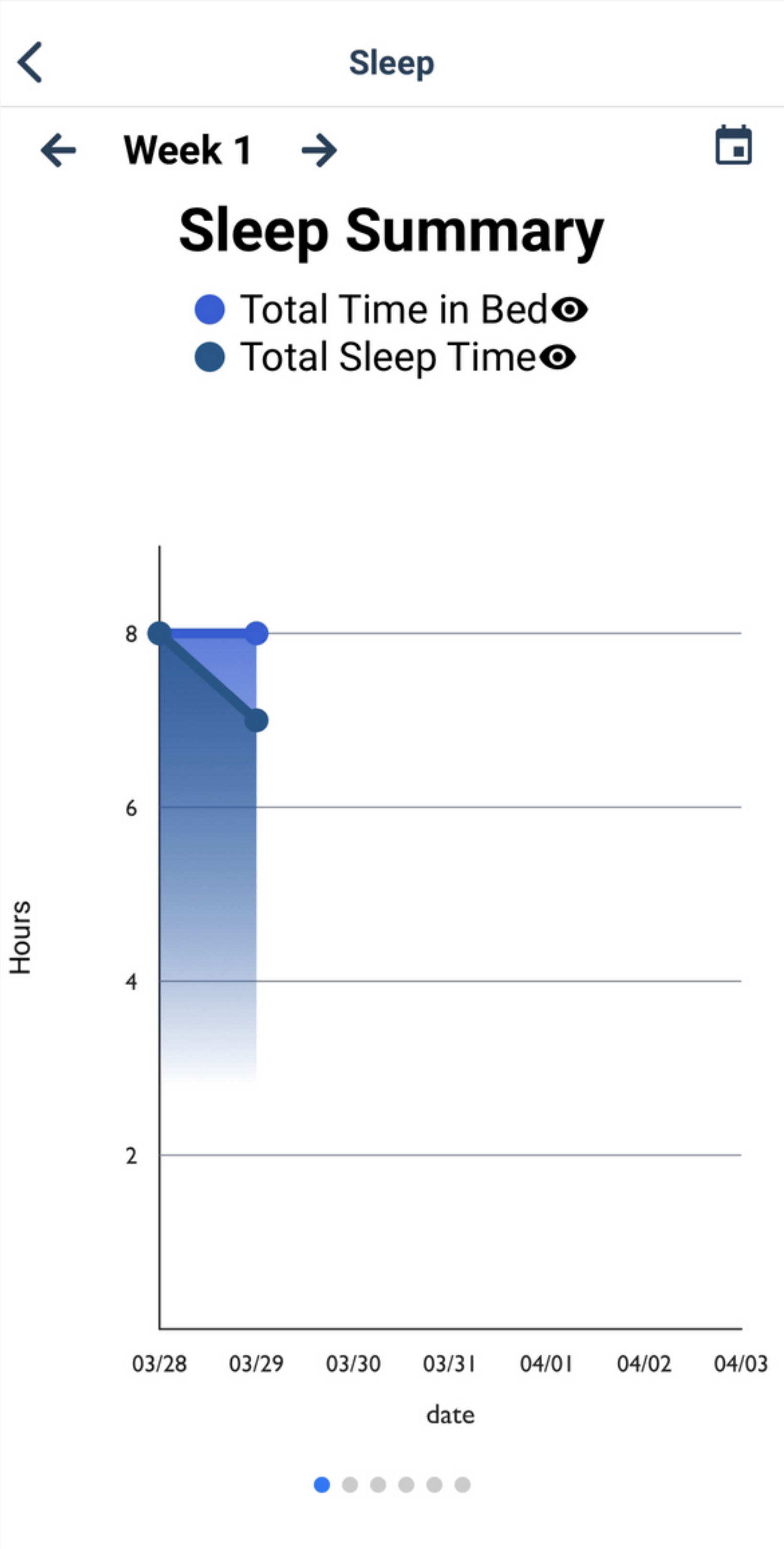

SLEEP PRESCRIPTION

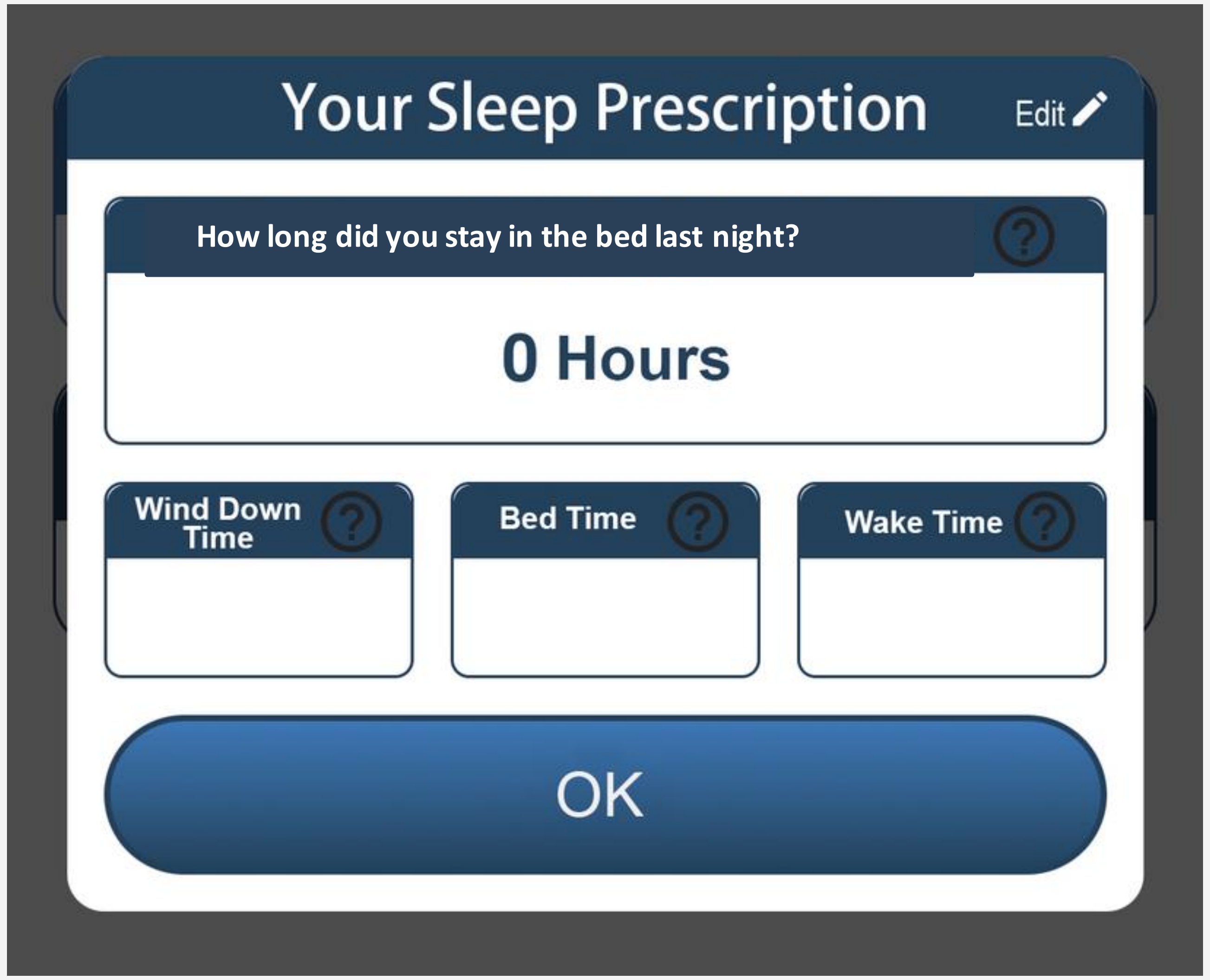

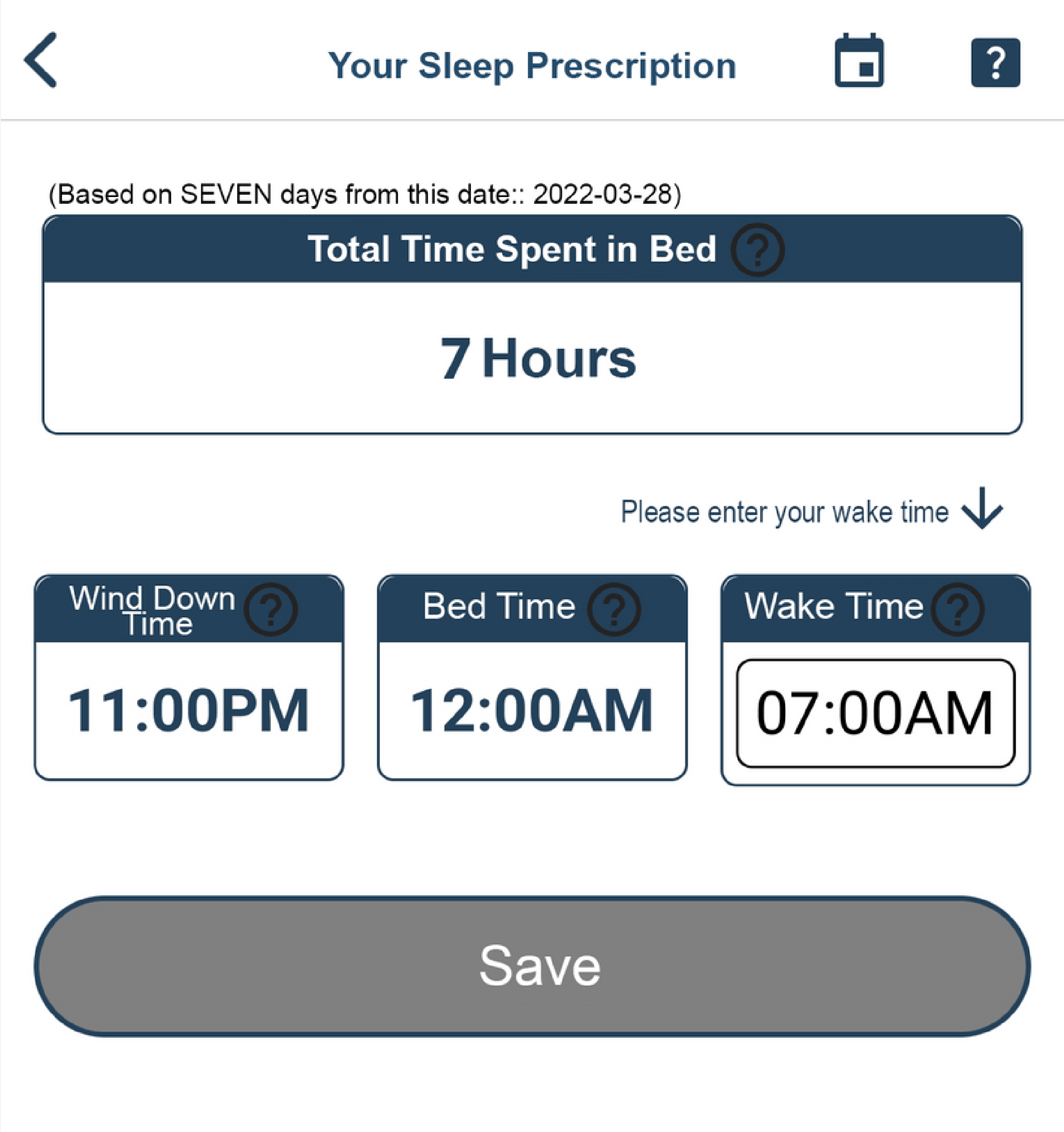

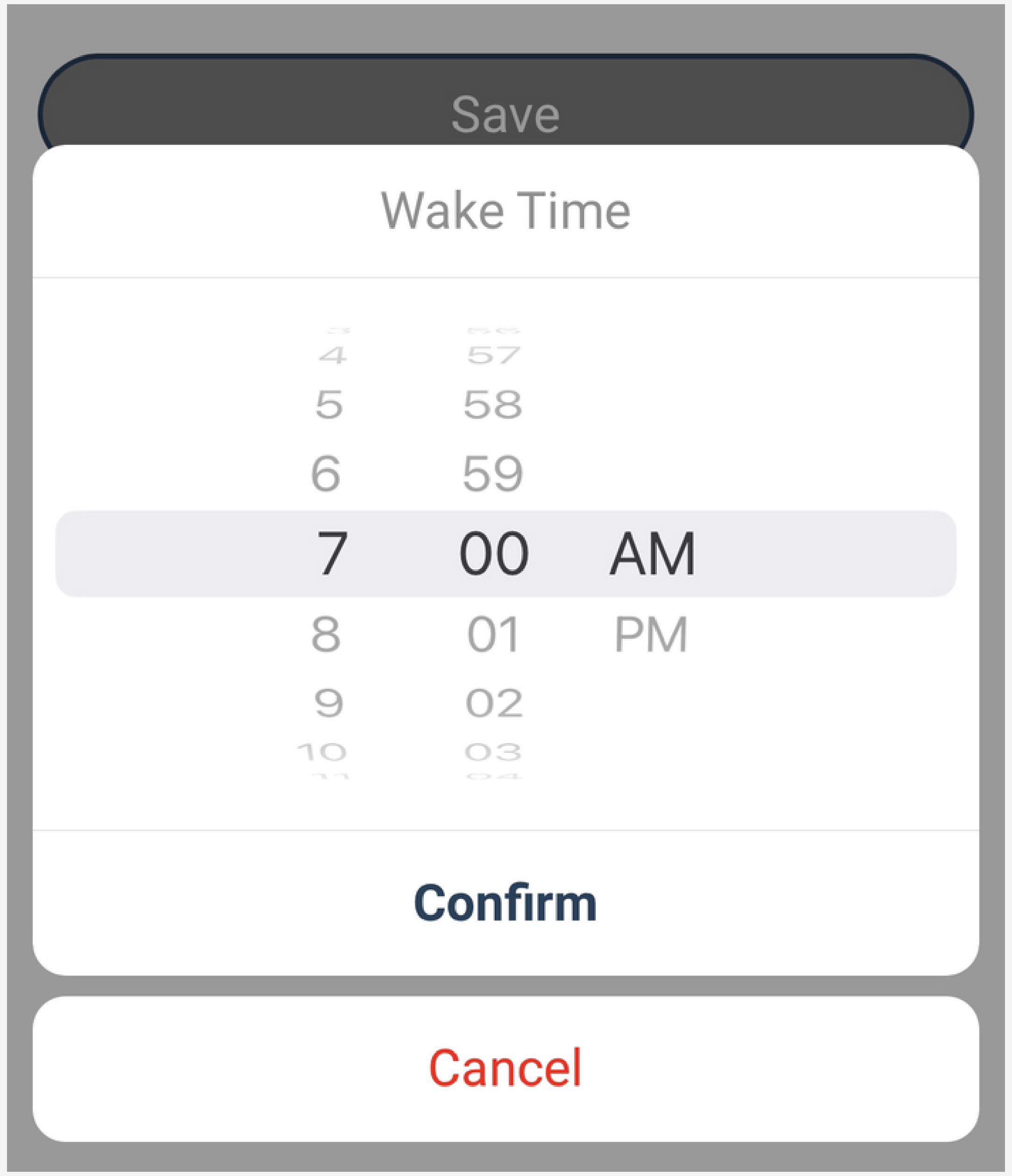

RESOURCE LIBRARY (INFOGRAPHICS & VIDEOS)

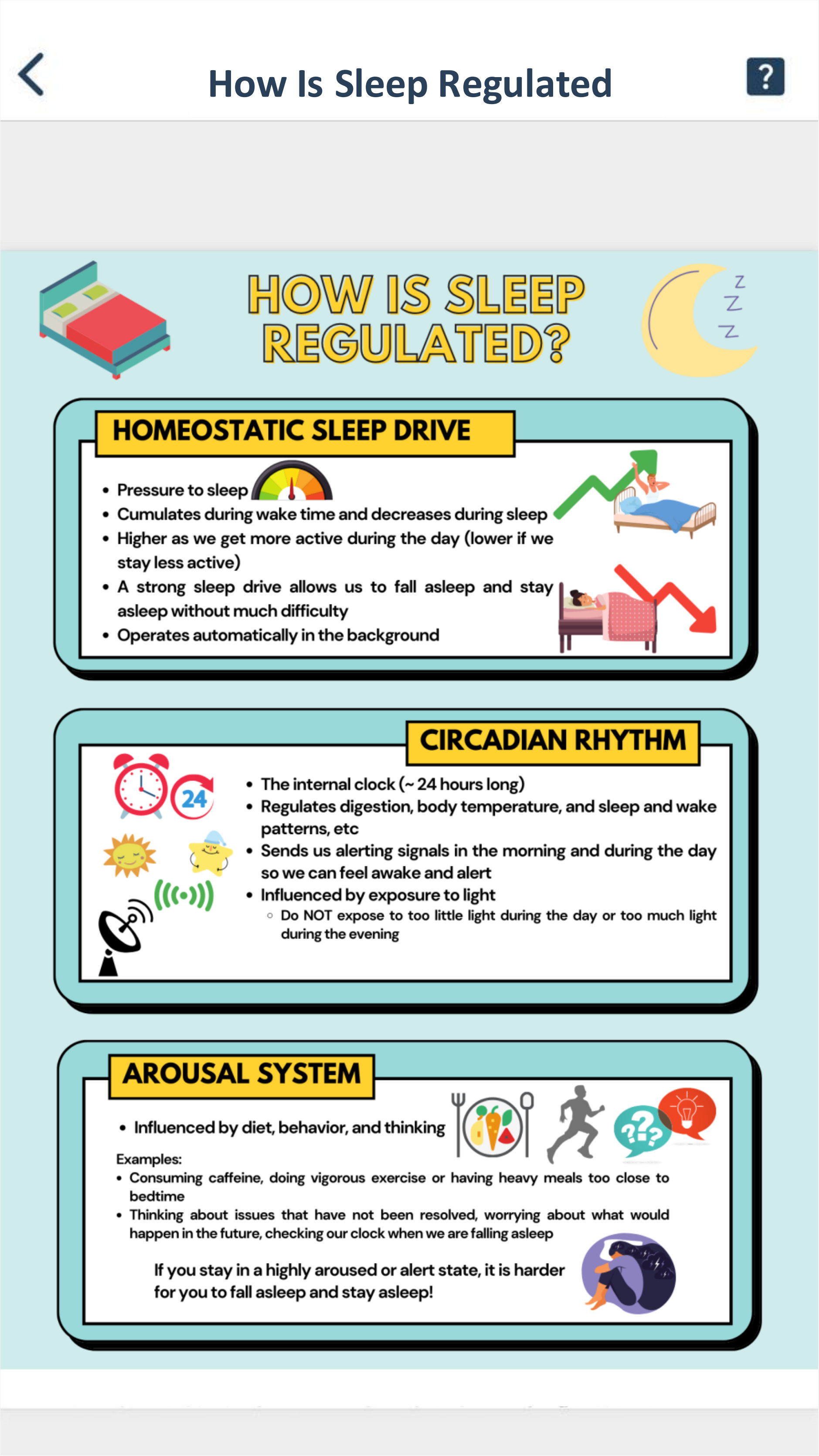

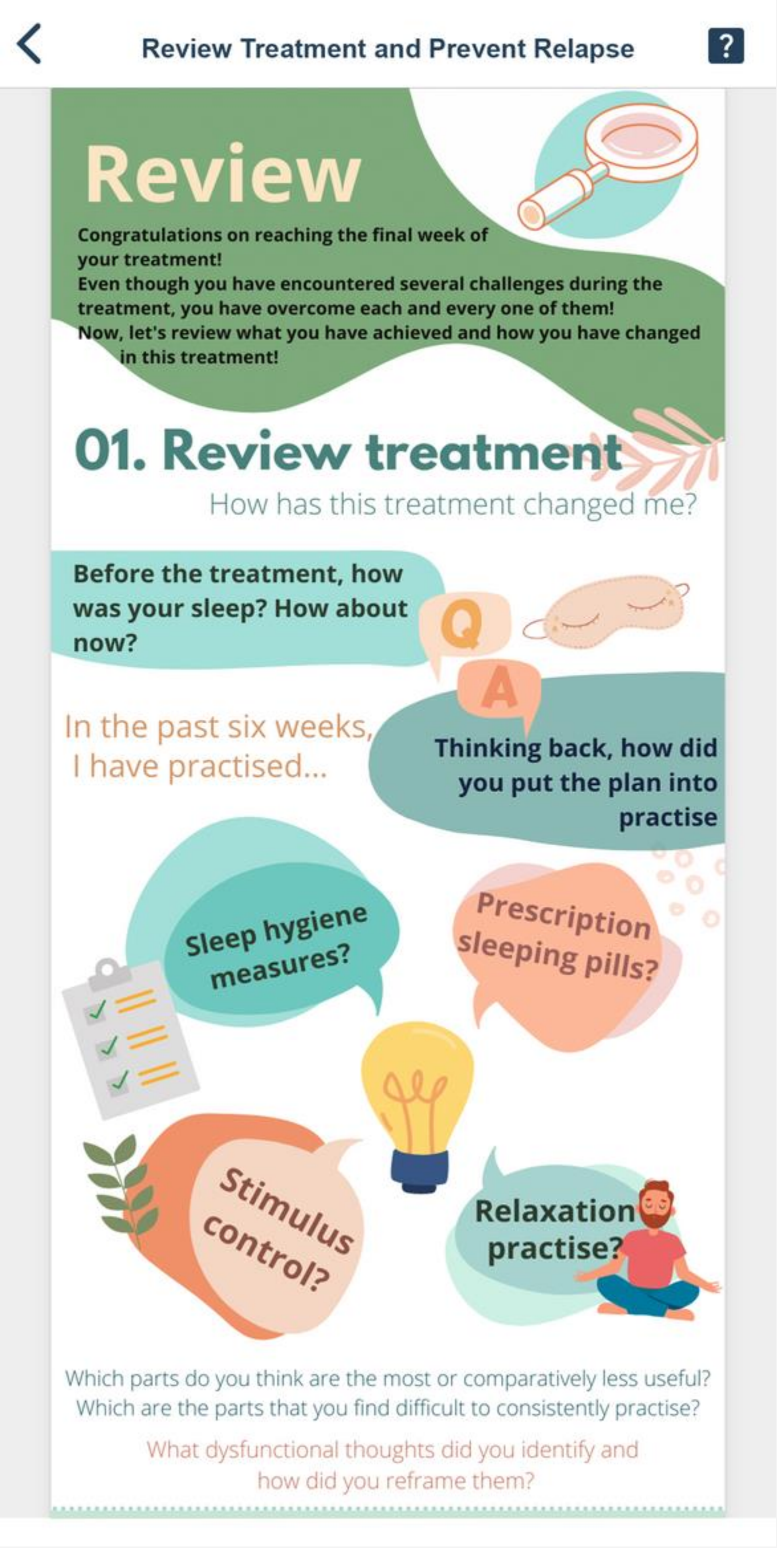

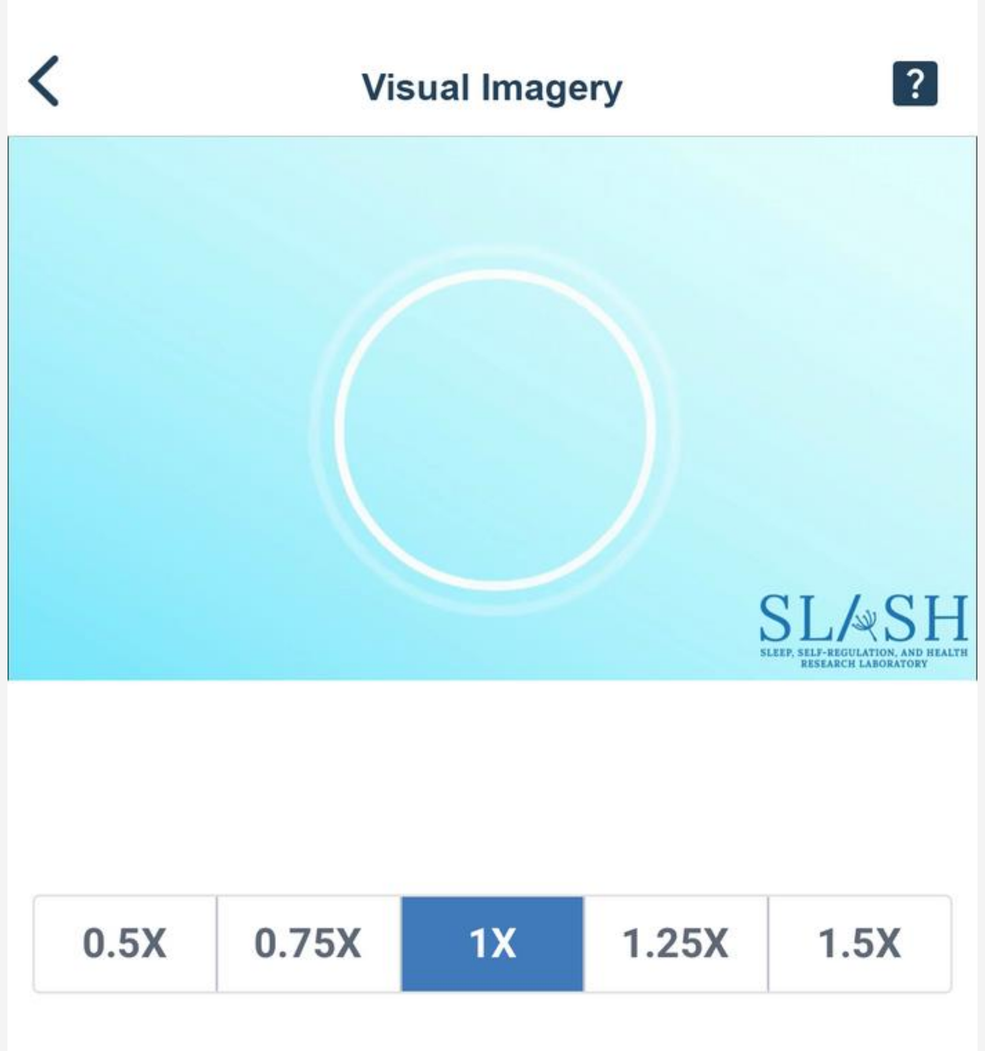

Supplement: Multimedia Appendix 1 [file mental_v11i1e51716_app1.pdf]
